# A Formal Synthesis of (+)-Hannokinol Using a Chiral Horner–Wittig Reagent

**DOI:** 10.3390/molecules29153710

**Published:** 2024-08-05

**Authors:** Michael Tapera, Federica Borghi, Jan Lukas Mayer-Figge, Fabia Mittendorf, Ibrahim-Ethem Celik, Adrián Gómez-Suárez, Stefan F. Kirsch

**Affiliations:** Organic Chemistry, Bergische Universität Wuppertal, Gaußstr. 20, 42119 Wuppertal, Germany

**Keywords:** total synthesis, Horner–Wittig, 1,3-diols

## Abstract

Herein, we report a concise and efficient formal synthesis of (+)-hannokinol. Key to this new strategy is the use of a chiral Horner–Wittig reagent, readily available from 2-deoxy-D-ribose, to introduce the chiral 1,3-diol motif.

## 1. Introduction

**Scheme 1:**
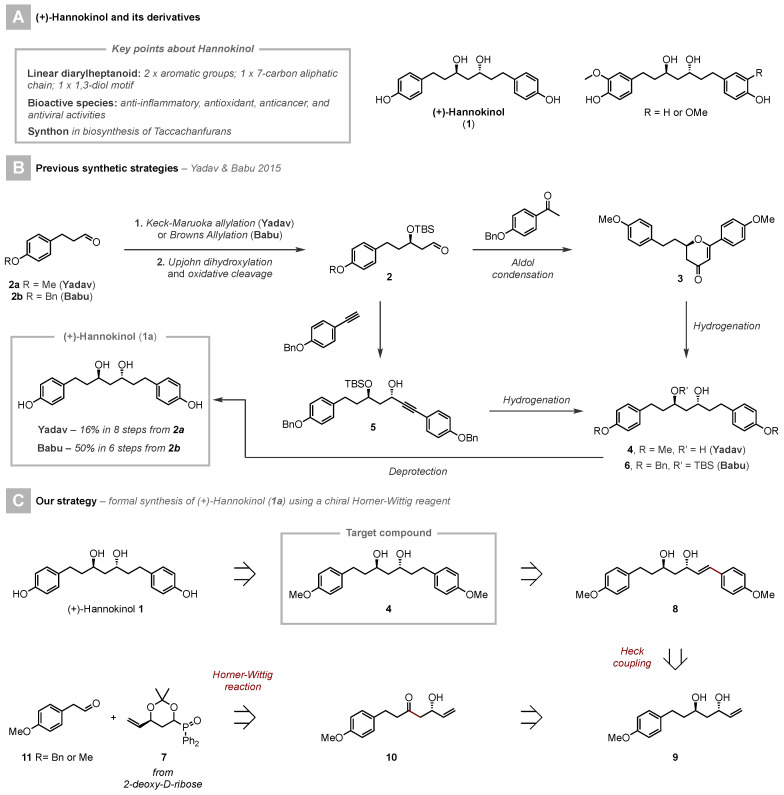
(+)-Hannokinol: properties, synthetic routes, and our approach. (**A**) (+)-Hannokinol structure and importance. (**B**) Reported synthesis of (+)-Hannokinol [11]. (**C**) Our strategy for the synthyesis of (+)-Hannokinol using a chiral Horner-Wittig reagent.

(+)-Hannokinol (**1**) is a linear diarylheptanoid that belongs to a group of naturally occurring compounds characterized by two aromatic rings connected by a seven-carbon aliphatic chain containing a 1,3-diol moiety (Scheme 1A) [1,2]. It was first isolated from the seeds of *Alpinia blepharocalyx* in 1995, and it was identified with a variety of potent biological activities, including anti-inflammatory, antioxidant, anticancer, and antiviral [3,4,5,6,7,8,9]. In 2002, it was also extracted from the rhizomes of *Tacca chantrieri*, which are used in traditional Chinese medicine to treat gastric ulcers, enteritis, and hepatitis [3]. Furthermore, it has been hypothesized that it can act as a synthon during the biosynthesis of other natural products, such as taccachanfurans [10].

In 2015, the first total syntheses of **1** were independently reported by Yadav and Babu following similar synthetic routes (Scheme 1B). Yadav and coworkers reported the total synthesis of (+)-hannokinol [11], using compound **2** as the key intermediate in their strategy. This species can be readily accessed from known aldehyde **2a** via Keck–Maruoka allylation [12,13], followed by Upjohn dihydroxylation [14] and oxidative cleavage. Next, compound **2** undergoes aldol addition followed by oxidation and TBS removal, which cyclizes to tetrahydropyran **3**. Selective hydrogenation of **3** produces intermediate **4**, which, upon deprotection, affords (+)-hannokinol in 16% yield after eight steps from aldehyde **2a**. A few months later, Babu and coworkers reported a similar strategy for the synthesis of (+)-hannokinol from compound **2** [15]. In this case, aldehyde **2** was prepared from **2b** via Brown’s asymmetric allylation [9], followed by Upjohn dihydroxylation [14] and oxidative cleavage. The key step in Babu’s route was a diethylzinc (Et_2_Zn)-mediated diastereoselective alkynylation of **2** to access alkyne **5**, which, after hydrogenation, affords intermediate **6**. Deprotection of the latter afforded (+)-hannokinol in 50% yield after six steps from aldehyde **2b**. Due to its compelling biological activities and its potential use as a natural building block, (+)-hannokinol has captured the interest of organic and medicinal chemists. Therefore, the development of alternative and scalable synthetic strategies from readily available starting materials is of high interest.

Herein, we present our strategy to access diol intermediate **4**, leading to the formal total synthesis of **1** (Scheme 1C). Key to our approach is the use of our modular strategy for the construction of 1,3-diols using chiral building block **7** [16,17], which is prepared from commonly accessible and inexpensive 2-deoxy-D-ribose [18]. In recent years, we have demonstrated the application of this reagent to the synthesis of complex natural products, such as bastimolide [19], aureosurfactin [20], cryptoconcatone D [18], and harzialactone A [21]. During our retrosynthetic analysis to access intermediate **4**, we envisioned the construction of the core linear diarylheptanoid skeleton via reduction of olefin **8**, which could be readily built via a Heck coupling from compound **9**. The latter could be synthesized through an Evans–Tishchenko *anti*-reduction of β-hydroxy-ketone **10** [22,23,24], which can be readily accessed from **7** and aldehyde **11** using a Horner−Wittig reaction.

## 2. Results and Discussion

### 2.1. Initial Approach towards the Total Synthesis of (+)-Hannokinol

**Scheme 2:**
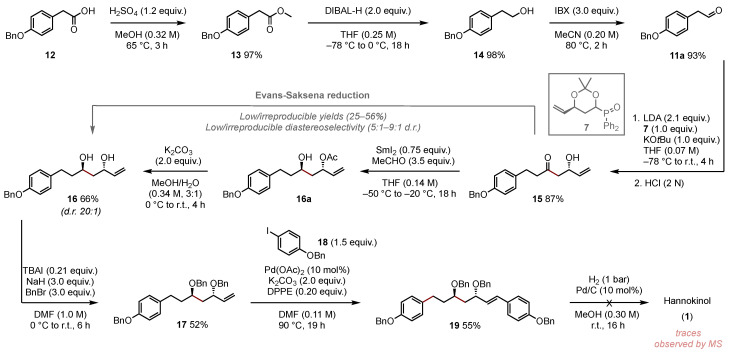
First approach towards the total synthesis of (+)-hannokinol using chiral Horner–Wittig reagent **7** and Heck coupling as key steps.

Our initial approach towards the synthesis of (+)-hannokinol (**1**) started with the conversion of commercially available benzyl-protected 4-hydroxyphenyl acetic acid (**12**) to the corresponding methyl ester **13** using classical Fischer esterification conditions (Scheme 2) [25]. The subsequent reduction of **13** using di*iso*butylaluminium hydride (DIBAL-H) afforded primary alcohol **14** in 98% yield. The latter could be selectively oxidized to the corresponding aldehyde using 2-iodoxybenzoic acid (IBX) in acetonitrile at 80 °C, affording **11a** in 93% yield [26]. Next, we proceeded to build the key heptanoid chain and the *anti*-1,3-diol motif. Using a robust Horner–Wittig reaction involving aldehyde **11a** and the chiral building block **7** (readily prepared in 7 steps from 2-deoxy-D-ribose) [20], β-hydroxy-ketone **15** was obtained in 87% yield. To access the *anti*-1,3-diol motif, we considered two strategies: (A) direct reduction of **15** to access *anti*-1,3-diol **16** using Evans–Saksena conditions [27] or (B) reduction of **15** under Evans–Tishchenko conditions [21,28] followed by deprotection of the resulting acetate-protected product to access *anti*-1,3-diol **16**. Unfortunately, the most direct reduction using Evans–Saksena conditions afforded irreproducible yields (25–56%) and diastereoselectivities (*d.r.* 5:1–9:1). Therefore, we proceeded with strategy B: Evans–Tishchenko reduction, followed by deprotection. Synthesis of *anti*-1,3-diol monoester **16a** was achieved by directed *anti*-reduction using samarium (II) iodide (SmI_2_) and acetaldehyde in THF from –50 °C to –20 °C for 18 h and used in the next step without purification. Subsequent deprotection under basic conditions [29] afforded the targeted *anti*-1,3-diol **16** in 66% yield over two steps and excellent diastereoselectivity (*d.r*. > 20:1). Next, the diol motif was converted to the corresponding dibenzyl protected species **17** in 52% yield using benzyl bromide in the presence of tetra-*n*-butylammonium iodide (TBAI) and sodium hydride (NaH). After the protection step, **17** was subjected to Pd-catalyzed Heck coupling [20] with benzyl-protected 4-iodophenol **18**. This afforded the key intermediate **19** in 55% yield. The last step of our strategy was the simultaneous reduction of the alkene in **19** and the global benzyl deprotection of the phenol core and *anti*-1,3-diol motif to access (+)-hannokinol (**1**). Unfortunately, using 10 mol% of Pd/C under a hydrogen atmosphere (1 bar) afforded only a trace amount of **1**, which was observed using mass spectrometry. We hypothesized that failure at this stage could be due to inefficient benzyl deprotection, as the benzyl moiety is susceptible to aromatic saturation, resulting in low yields of deprotected products [30,31].

### 2.2. Change of Strategy: Synthesis of Intermediate **4**, Formal Synthesis of (+)-Hannokinol

**Scheme 3:**
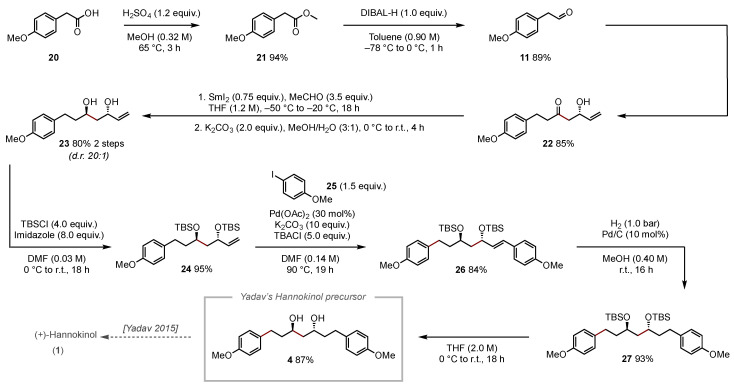
Synthesis of key intermediate **4** using a chiral Horner–Wittig reagent and Heck coupling as key steps [11].

Since the simultaneous global benzyl deprotection/olefin reduction step proved to be more difficult than initially anticipated, we decided to slightly alter our strategy. We realized that changing the protecting groups at both the aromatic rings and the diol unit would enable us to use our strategy to access intermediate **4**, thus intercepting Yadav’s route for the synthesis of **1** (Scheme 3).

Our new strategy began with the conversion of commercially available 4-methoxyphenylacetic acid **20** to the corresponding methyl ester **21** (94% yield). Carefully controlling the reduction of **21**, following a procedure reported by Tao using DIBAL-H [32], enabled the selective reduction of the ester moiety to the corresponding aldehyde **11** in 89% yield. Next, the key Horner–Witting reaction of **11** with chiral building block **7** furnished β-hydroxy-ketone **22** in 85% yield. Subsequent a*nti*-reduction under Evans–Tishchenko conditions and deprotection afforded *anti*-1,3-diol **23** in high yields (80%) and high stereoselectivity (*d.r.* > 20:1). Protection of the 1,3-diol motif using *tert*-butyldimethylsilylchloride (TBSCl) and imidazole furnished the corresponding *bis*-TBS-protected species **24** in 95% yield. The latter was then subjected to Pd-catalyzed Heck cross coupling with iodide **25** following a modified procedure reported by Kalesse [33]. Gratifyingly, the reaction proceeded smoothly, affording compound **26** in 84% yield. Subsequent hydrogenation of the alkene motif in **26** using 10 mol% Pd/C under 1.0 bar of hydrogen afforded the corresponding alkane **27** in nearly quantitative yields (93%). Finally, deprotection of the silyl ethers with HF·pyridine complex [34] afforded **4** in excellent yields (87%), thus intercepting the synthetic route for the preparation of (+)-hannokinol (**1**) reported by Yadav and coworkers [11].

## 3. Materials and Methods

### 3.1. General Remarks

The commercially available reagents and solvents were used as purchased. Thin layer chromatography (TLC) was conducted with precoated aluminum sheets (silica gel 60 F254) and visualized by exposure to UV light (254 nm) or staining with potassium permanganate (KMnO_4_) or ceric ammonium molybdate (CAM) and subsequent heating. Flash column chromatography was performed on silica gel (40–60 µm); the eluent used is reported in the respective experiments. Abbreviations for solvents and chemicals are as follows: CyHex: cyclohexane, EA: ethyl acetate, THF: tetrahydrofuran, DCM: dichloromethane, MeOH: methanol, DMF: N,N-dimethylformamide, DE: diethyl ether, PE: petroleum ether, and MeCN: acetonitrile. IR spectra were measured using the ATR technique in the range of 400–4000 cm^−1^. ^1^H NMR spectra were recorded with 600 MHz or 400 MHz instruments from Bruker, (Billerica, MA, USA), ^13^C NMR spectra at 151 MHz or 101 MHz, and ^19^F NMR spectra at 376 MHz. Chemical shifts are reported in ppm relative to the solvent signal and coupling constants J in Hz. Multiplicities were defined by standard abbreviations. High-resolution mass spectra (HRMS) were obtained using ESI ionization (positive) on a Bruker micrOTOF. The optical rotation of unknown chiral substances were measured using a Krüss Optronic at 584 nm wavelength. The temperature during measurements was kept at 20 °C with a defined concentration (g/100 mL) in the described solvent.

### 3.2. Synthesis and Charaecterization of Compounds

#### 3.2.1. Methyl 2-(4-(Benzyloxy)phenyl)acetate (**13**)

To a solution of 2-(4-(benzyloxy)phenyl)acetic acid **12** (5.00 g, 20.6 mmol, 1.0 equiv.) in MeOH (65 mL) was added conc. H_2_SO_4_ (1.33 mL, 1.2 equiv.). The reaction mixture was heated under reflux (65 °C) for 3 h. After this time, the reaction mixture was allowed to cool down to room temperature, concentrated in vacuo, and dissolved in EA. The resulting solution was dried with sodium sulfate and filtered. The filtrate was concentrated in vacuo to afford a methyl ester **13** (5.13 g, yield 97%) as a yellow oil. TLC: R_f_ = 0.44 (EA/CyHex = 3:7) [KMnO_4_]. ^1^H NMR (600 MHz, CDCl_3_) δ 7.43 (d, *J* = 7.5 Hz, 2H), 7.39 (d, *J* = 15.0 Hz, 2H), 7.33 (t, *J* = 7.3 Hz, 1H), 7.20 (d, *J* = 8.8 Hz, 2H), 6.94 (d, *J* = 8.7 Hz, 2H), 5.06 (s, 2H), 3.69 (s, 3H), 3.57 (s, 2H). ^13^C NMR (151 MHz, CDCl_3_) δ 172.5, 158.1, 137.2, 130.4, 128.7, 128.1, 127.6, 126.5, 115.1, 70.2, 52.1, 40.5. HRMS (ESI) *m*/*z*: [M + Na^+^] calcd. for C_16_H_16_NaO_3_: 279.0991; found: 279.0992. All the characterization data are consistent with the literature [25].

#### 3.2.2. 2-(4-(Benzyloxy)phenyl)ethan-1-ol (**14**)

To a solution of methyl 2-(4-(benzyloxy)phenyl)acetate **13** (3.70 g, 14.4 mmol, 1.0 equiv.) in anhydrous THF (58 mL) was added dropwise DIBAL-H (1.0 M solution in toluene, 28.9 mL, 28.9 mmol, 2.0 equiv.) at −78 °C. After the addition, the cold bath was removed, and the reaction mixture was stirred at room temperature for 7 h. Glycerol (8 mL) and K/Na-tartrate solution (20 mL) were then added at 0 °C. The mixture was stirred overnight at room temperature and then extracted three times with EA. The combined organic phase was washed with a sodium bicarbonate solution, dried with sodium sulfate, filtered, and concentrated under reduced pressure. The crude residue was purified by silica gel column chromatography (EA/CyHex = 3:7) to give product **14** (3.26 g, yield 98%) as a colorless solid. TLC: R_f_ = 0.18 (EA/CyHex = 3:7) [KMnO_4_]. ^1^H NMR (600 MHz, CDCl_3_) δ 7.43 (d, *J* = 7.6 Hz, 2H), 7.39 (t, *J* = 7.5 Hz, 2H), 7.32 (t, *J* = 7.3 Hz, 1H), 7.15 (d, *J* = 8.6 Hz, 2H), 6.94 (d, *J* = 8.6 Hz, 2H), 5.06 (s, 2H), 3.83 (t, *J* = 6.5 Hz, 2H), 2.82 (t, *J* = 6.5 Hz, 2H), 1.43 (br, 1H). ^13^C NMR (151 MHz, CDCl_3_) δ 157.7, 137.3, 130.9, 130.1, 128.7, 128.1, 127.6, 115.2, 70.2, 64.0, 38.5. All the characterization data are consistent with the literature [25].

#### 3.2.3. 2-(4-(Benzyloxy)phenyl)acetaldehyde (**11a**)

To a solution of 2-(4-(benzyloxy)phenyl)ethan-1-ol **14** (4.00 g, 17.5 mmol, 1.0 equiv.) in dry acetonitrile (88 mL) was added IBX (13.0 g, 52.6 mmol, 3.0 equiv.), and the suspension was stirred at 80 °C for 2 h and then cooled to room temperature. The suspension formed after cooling was filtered through a pad of celite and washed with EA. The filtrate was washed with a sodium bicarbonate solution, and the aqueous phase was extracted with EA (3 × 50 mL). The combined organic phases were dried over sodium sulfate and the solvent was removed under reduced pressure. The crude residue was purified by column chromatography (EA/CyHex = 2:8 → 3:7) to give product **11a** (3.69 g, yield 93%) as a yellowish solid. TLC: R_f_ = 0.63 (EA/CyHex = 1:5) [KMnO_4_]. ^1^H NMR (400 MHz, CDCl_3_) δ 9.73 (t, *J* = 2.4 Hz, 1H), 7.47–7.38 (m, 4H), 7.36–7.30 (m, 1H), 7.14 (d, *J* = 8.7 Hz, 2H), 6.98 (d, *J* = 8.7 Hz, 2H), 5.07 (s, 2H), 3.63 (d, *J* = 2.4 Hz, 2H). ^13^C NMR (101 MHz, CDCl_3_) δ 199.8, 158.3, 137.0, 130.8, 128.8, 128.2, 124.1, 115.5, 70.2, 49.9. IR (ATR): $\tilde{\nu }$ [cm^−1^] = 3032, 2868, 1719, 1610, 1583, 1509, 1454, 1238, 1176, 1014, 927, 861, 612, 593. HRMS (ESI) *m*/*z*: [M + Na^+^] calcd. for C_15_H_14_NaO_2_: 249.0886; found: 249.0886. All the characterization data are consistent with the literature [25].

#### 3.2.4. (*S*)-1-(4-(Benzyloxy)phenyl)-5-hydroxyhept-6-en-3-one (**15**)

To a solution of diisopropylamine (2.15 mL, 15.3 mmol, 2.1 equiv.) in dry THF (73 mL) was added *n*-butyllithium (2.5 M in hexane, 6.13 mL, 15.3 mmol, 2.1 equiv.) at −78 °C. After the cooling was removed for 15 min, (*S*)-building block (2.50 g, 7.30 mmol, 1.0 equiv.) dissolved in dry THF (24 mL) was added slowly at −78 °C. The solution was allowed to stir for 1 h at this temperature. Following the addition of 2-(4-(benzyloxy)phenyl)acetaldehyde **11a** (3.50 g, 15.5 mmol, 3.0 equiv.) dissolved in dry THF (9 mL), the reaction was allowed to warm to rt within 1 h. After the addition of potassium *tert*-butoxide (863 mg, 7.30 mmol, 1.0 equiv.), the reaction mixture was stirred for one more hour at room temperature and was quenched with a saturated aqueous ammonium chloride solution (30 mL), and the aqueous layer was extracted with dichloromethane (3 × 50 mL). The combined organic layers were washed with hydrochloric acid (2 M, 2 × 50 mL), and the aqueous layer was again extracted with dichloromethane (3 × 50 mL). The combined organic layers were washed with brine (100 mL), dried with sodium sulfate, and filtered. The filtrate was concentrated in vacuo, and the crude residue was purified by silica gel column chromatography (EA/CyHex = 3:7) to give the product **15** (1.97 g, yield 87%) as a light-yellow oil. TLC: R_f_ = 0.21 (EA/CyHex = 3:7) [KMnO_4_]. ^1^H NMR (400 MHz, CDCl_3_) δ 7.51–7.34 (m, 5H), 7.35–7.28 (m, 1H), 7.09 (d, *J* = 8.7 Hz, 2H), 6.90 (d, *J* = 8.6 Hz, 2H), 5.84 (ddd, *J* = 17.2, 10.5, 5.5 Hz, 1H), 5.28 (dt, *J* = 17.2, 1.4 Hz, 1H), 5.12 (dt, *J* = 10.5, 1.4 Hz, 1H), 5.04 (s, 2H), 4.56 (dtt, *J* = 7.0, 5.5, 1.5 Hz, 1H), 2.92–2.80 (m, 2H), 2.74 (ddd, *J* = 8.2, 6.8, 1.1 Hz, 2H), 2.62 (d, *J* = 5.5 Hz, 2H). ^13^C NMR (101 MHz, CDCl_3_) δ 210.4, 157.5, 139.2, 137.3, 133.2, 129.4, 128.7, 128.1, 127.6, 70.3, 68.8, 49.1, 45.5, 28.8. IR (ATR): $\tilde{\nu }$ [cm^−1^] = 3351, 3281, 3091, 3059, 3037, 3008, 2929, 2902, 2872, 1705, 1644, 1610, 1580, 1510, 1465, 1454, 1418, 1410, 1378, 1340, 1322, 1297, 1239, 1174, 1148, 1117, 1096, 1045, 1010, 994, 976, 917, 860, 845, 820, 807, 782, 747. [α]^20^_D_ = −9.5 (c = 1.0, DCM). HRMS (ESI) *m*/*z*: [M + Na^+^] calcd. for C_20_H_22_NaO_3_: 333.1465; found: 333.1461.

#### 3.2.5. (3*S*,5*R*)-7-(4-(Benzyloxy)phenyl)hept-1-ene-3,5-diol (**16**)

To a solution of (*S*)-1-(4-(benzyloxy)phenyl)-5-hydroxyhept-6-en-3-one **15** (530 mg, 1.71 mmol, 1.0 equiv.) in dry THF (12 mL) was added acetaldehyde (337 µL, 5.98 mmol, 3.5 equiv.). At −50 °C, SmI_2_ (0.1 M in THF, 12.8 mL, 1.28 mmol, 0.75 equiv.) was added slowly under the exclusion of light. The reaction mixture was allowed to stir at −20 °C for 18 h. After the addition of a saturated aqueous sodium bicarbonate solution (15 mL), the layers were separated, and the aqueous layer was extracted with EA (3 × 10 mL). The combined organic layers were washed with brine (20 mL), dried with sodium sulfate, and filtered. The filtrate was concentrated in vacuo, and the crude residue was purified by column chromatography (EA/CyHex = 2:8) to give the product **16** (563 mg, yield 93%) as a colorless oil.

To a solution of (3*S*,5*R*)-7-(4-(benzyloxy)phenyl)-5-hydroxyhept-1-en-3-yl acetate (500 mg, 1.41 mmol, 1.0 equiv.) in MeOH/H_2_O (4,2 mL, 3:1) was added potassium carbonate (389 mg, 2.82 mmol, 2.0 equiv.) at 0 °C. The reaction mixture was stirred for 4 h at 0 °C and then allowed to warm up to room temperature. Upon completion of the reaction indicated by TLC analysis, H_2_O (10 mL) and ethyl acetate (10 mL) were added, the layers were separated, and the aqueous layer was extracted with ethyl acetate (3 × 10 mL). The combined organic layers were washed with brine (15 mL), dried with sodium sulfate, and filtered. The filtrate was concentrated in vacuo, and the crude residue was purified by column chromatography (EA/CyHex = 3:7) to give the product **16** (313 mg, yield 71%) as a colorless oil. TLC: R_f_ = 0.29 (EA/CyHex = 1:1) [KMnO_4_]. ^1^H NMR (600 MHz, CDCl_3_) δ 7.43 (d, *J* = 7.5 Hz, 1H), 7.38 (t, *J* = 7.6 Hz, 5H), 7.32 (t, *J* = 7.3 Hz, 2H), 7.11 (d, *J* = 8.5 Hz, 5H), 6.90 (d, *J* = 8.6 Hz, 5H), 5.93 (ddd, *J* = 17.3, 10.5, 5.5 Hz, 3H), 5.29 (d, *J* = 17.2 Hz, 2H), 5.15 (s, 1H), 5.04 (s, 6H), 4.48 (dt, *J* = 5.4, 1.7 Hz, 2H), 3.97 (dd, *J* = 4.7, 3.2 Hz, 2H), 2.73 (ddd, *J* = 13.8, 9.8, 5.7 Hz, 3H), 2.62 (ddd, *J* = 13.9, 9.7, 6.4 Hz, 3H), 2.02 (s, 9H), 1.90–1.56 (m, 12H). ^13^C NMR (151 MHz, CDCl_3_) δ 157.1, 140.7, 137.2, 134.2, 129.3, 128.6, 127.9, 127.5, 114.9, 114.5, 70.8, 70.1, 68.7, 42.3, 39.4, 31.2. [α]^20^_D_ = 16.8 (c = 1.0, DCM). IR (ATR): $\tilde{\nu }$ [cm^−1^] = 3286, 3090, 3066, 3035, 3015, 2928, 2906, 2852, 1646, 1611, 1582, 1511, 1422, 1402, 1382, 1349, 1312, 1296, 1278, 1240, 1173, 1134, 1110, 1069, 1038, 1026, 1014, 1001, 991, 975, 918, 903, 863, 837, 825, 808, 781, 734, 696. HRMS (ESI) *m*/*z*: [M + Na^+^] calcd. for C_20_H_24_NaO_3_: 335.1620; found: 335.1618.

#### 3.2.6. ((((3*S*,5*R*)-7-(4-(Benzyloxy)phenyl)hept-1-ene-3,5-diyl)bis(oxy))bis(methylene))dibenzene (**17**)

To a solution of (3*S*,5*R*)-7-(4-(benzyloxy)phenyl)hept-1-ene-3,5-diol **16** (34.9 mg, 109 µmol, 1.0 equiv.) in 1.0 mL of dry DMF was added tetra-*n*-butylammonium iodide (8.40 mg, 22.9 µmol, 0.20 equiv.) and NaH (13.1 mg, 60% in mineral oil, 327 µmol, 3.0 equiv.). The reaction mixture was stirred for 30 min at 0 °C. Then, benzyl bromide (39.0 µL, 56.2 mg, 328 µmol, 3.0 equiv.) was added to the mixture and stirred for 5 h at room temperature under argon. After reaction completion, the crude mixture was directly purified by column chromatography (EA/CyHex = 1:9 → 2:8). A further separation was carried out using preparative HPLC (MeCN:H_2_O = 9:1) to give product **17** (27.7 mg, yield 52%) as a colorless solid. TLC: R_f_ = 0.54 (EA/CyHex = 2:8) [KMnO_4_]. ^1^H NMR (400 MHz, CDCl_3_) δ 7.46 (d, *J* = 7.1 Hz, 2H), 7.40 (t, *J* = 7.3 Hz, 2H), 7.37–7.26 (m, 11H), 7.08 (d, *J* = 8.6 Hz, 2H), 6.90 (d, *J* = 8.6 Hz, 2H), 5.79 (ddd, *J* = 17.5, 10.3, 7.5 Hz, 1H), 5.31–5.19 (m, 2H), 5.06 (s, 2H), 4.58 (d, *J* = 11.6 Hz, 1H), 4.50 (d, *J* = 11.4 Hz, 1H), 4.35 (d, *J* = 11.4 Hz, 1H), 4.24 (d, *J* = 11.6 Hz, 1H), 4.01 (td, *J* = 8.0, 4.5 Hz, 1H), 3.79–3.68 (m, 1H), 2.64 (t, *J* = 8.0 Hz, 2H), 1.94–1.73 (m, 4H). ^13^C NMR (101 MHz, CDCl_3_) δ 157.0, 139.1, 138.9, 138.7, 134.8, 129.3, 128.6, 128.3, 127.9 (d, *J* = 1.7 Hz), 127.9, 127.5 (d, *J* = 4.1 Hz), 116.7, 114.8, 75.0, 71.2, 70.3, 70.1, 41.2, 36.3, 30.4. IR (ATR): $\tilde{\nu }$ [cm^−1^] = 3087, 3063, 3030, 3007, 2860, 1610, 1583, 1509, 1496, 1453, 1420, 1380, 1355, 1310, 1298, 1237, 1175, 1089, 1067, 1026, 993, 924, 860, 820, 732, 694, 644, 613, 560, 532, 514, 458. [α]^20^_D_ = 16.6 (c = 1.0, DCM). HRMS (ESI) *m*/*z*: [M + Na^+^] calcd. for C_34_H_36_NaO_3_: 515.2564; found: 515.2567.

#### 3.2.7. 4,4′-((3*S*,5*R*,*E*)-3,5-*Bis*(benzyloxy)hept-1-ene-1,7-diyl)*bis*((benzyloxy)benzene) (**19**)

In a 4 mL vial was added palladium (II) acetate (1.23 mg, 5.48 µmol, 10 mol%), DPPE (4.37 mg, 11.0 µmol, 20 mol%), potassium carbonate (15.1 mg, 110 µmol, 2.0 equiv.), and 100 µL of dry DMF. A solution of alkene **17** (27.0 mg, 54.8 mmol, 1.0 equiv.) and 1-benzyloxy-4-iodobenzene **18** (25.5 mg, 82.2 µmol, 1.5 equiv.) in 700 µL of dry DMF was added dropwise. The reaction mixture was stirred for 18 h at 90 °C under argon. The solution was then cooled to room temperature and purified directly by column chromatography (EA:CyHex = 1:9). A small amount of the solid was dissolved in acetonitrile and separated via preparative HPLC (MeCN:H_2_O = 9:1). It was again purified by silica gel column chromatography (EA/CyHex = 1:9) to give **19** in the form of a slightly brownish solid (20.3 mg, yield 55%). TLC: R_f_ = 0.49 (EA/CyHex = 2:8) [KMnO_4_]. ^1^H NMR (600 MHz, CDCl_3_) δ 7.46–7.27 (m, 20H), 7.04 (dd, *J* = 15.7, 8.7 Hz, 4H), 6.94 (d, *J* = 8.8 Hz, 2H), 6.87 (d, *J* = 8.6 Hz, 2H), 6.48 (d, *J* = 15.9 Hz, 1H), 5.99 (dd, *J* = 15.9, 8.0 Hz, 1H), 5.12 (s, 1H), 5.08 (s, 1H), 5.03 (s, 2H), 4.59 (d, *J* = 11.7 Hz, 1H), 4.49 (d, *J* = 11.4 Hz, 1H), 4.35 (d, *J* = 11.3 Hz, 1H), 4.27 (d, *J* = 11.7 Hz, 1H), 4.12 (td, *J* = 8.5, 4.1 Hz, 1H), 3.81–3.67 (m, 1H), 2.63 (t, *J* = 8.0 Hz, 2H), 1.87 (m, 4H). ^13^C NMR (151 MHz, CDCl_3_) δ 158.7, 157.1, 139.0, 138.9, 137.4, 137.1, 134.9, 131.7, 129.5, 128.7 (d, *J* = 6.5 Hz), 128.5 (d, *J* = 2.6 Hz), 128.1 (d, *J* = 6.3 Hz), 128.0, 127.8, 127.7–127.5 (m), 115.2, 114.9, 75.1, 71.3, 70.3, 70.2 (d, *J* = 2.0 Hz), 41.6, 36.5, 30.5. IR (ATR): $\tilde{\nu }$ [cm^−1^] = 3088, 3063, 3031, 2924, 2858, 1729, 1606, 1583, 1509, 1454, 1381, 1297, 1240, 1174, 1070, 1026, 969, 913, 860, 819, 735, 696, 613, 512, 458. [α]^20^_D_ = −20.3 (c = 1.0, DCM). HRMS (ESI) *m*/*z*: [M + Na^+^] calcd. for C_47_H_46_NaO_4_: 697.3281; found: 697.3288.

#### 3.2.8. Methyl 2-(4-Methoxyphenyl)acetate (**21**)

To a solution of 4-methoxyphenylacetic acid **20** (6.00 g, 36.0 mmol, 1.0 equiv.) in MeOH (112 mL) was added conc. H_2_SO_4_ (2.32 mL, 43.3 mmol, 1.2 equiv.). The reaction mixture was heated under reflux (65 °C) for 3 h. The resulting mixture was cooled to room temperature, and the solvent was removed under vacuum. The crude residue was redissolved in EA, dried with sodium sulfate, filtered, concentrated, and purified by silica gel column chromatography (EA/CyHex = 1:1) to give the methyl ester (6.10 g, yield 94%) as a colorless oil. TLC: R_f_ = 0.82 (EA/CyHex = 1:1) [KMnO_4_]. ^1^H NMR (600 MHz, CDCl_3_) δ 7.20 (d, *J* = 8.6 Hz, 2H), 6.86 (d, *J* = 8.6 Hz, 2H), 3.79 (s, 3H), 3.69 (s, 3H), 3.57 (s, 2H). ^13^C NMR (151 MHz, CDCl_3_) δ 172.4, 158.9, 130.4, 126.2, 114.1, 55.4, 52.1, 40.4. IR (ATR): $\tilde{\nu }$ [cm^−1^] = 2952, 1732, 1612, 1511, 1299, 1242, 1151, 1012, 788. HRMS (ESI) *m*/*z*: [M + Na^+^] calcd. for C_10_H_12_NaO_3_: 203.0678; found: 203.0679. All the characterization data are consistent with the literature [32].

#### 3.2.9. 2-(4-Methoxyphenyl)acetaldehyde (**11**)

To a solution of methyl 2-(4-methoxyphenyl)acetate **21** (5.00 g, 27.8 mmol, 1.0 equiv.) in anhydrous toluene (70 mL) at −78 °C was added dropwise diisobutylaluminium hydride (1.2 M solution in toluene, 25.4 mL, 30.5 mmol, 1.1 equiv.). The reaction was stirred for 1 h. The reaction was quenched with MeOH (15 mL) and then poured into DCM, then washed sequentially with 1 M HCl (25 mL) and brine (25 mL). The organic phase was concentrated under reduced pressure. The crude residue was purified by silica gel column chromatography (DE/PE = 1:10) to give product **11** (3.70 g, yield 89%) as a colorless oil. TLC: R_f_ = 0.81 (DE/PE = 1:10) [KMnO_4_]. ^1^H NMR (400 MHz, CDCl_3_) δ 9.72 (t, *J* = 2.4 Hz, 1H), 7.13 (d, *J* = 8.8 Hz, 2H), 6.91 (d, *J* = 8.7 Hz, 2H), 3.81 (s, 3H), 3.63 (d, *J* = 2.4 Hz, 2H). ^13^C NMR (101 MHz, CDCl_3_) δ 200.1, 159.4, 131.1, 124.2, 114.9, 55.7, 50.2. IR (ATR): $\tilde{\nu }$ [cm^−1^] = 1721, 1611, 1511, 1245, 1177, 1032, 906, 825, 726, 648, 524. HRMS (ESI) *m*/*z*: [M + Na^+^] calcd. for C_9_H_10_NaO_2_: 173.0681; found: 173.0568. All the characterization data are consistent with the literature [35].

#### 3.2.10. 5-Hydroxy-1-(4-methoxyphenyl)hept-6-en-3-one (**22**)

To a solution of diisopropylamine (1.70 mL, 12.3 mmol, 2.1 equiv.) in dry THF (58 mL) was added *n*-butyllithium (2.5 M in hexane, 6.40 mL, 12.3 mmol, 2.1 equiv.) at −78 °C. After the cooling was removed for 15 min, (*S*)-building block **7** (2.00 g, 5.84 mmol, 1.0 equiv.) dissolved in dry THF (19 mL) was added slowly at −78 °C. The solution was allowed to stir for 1 h at this temperature. Following the addition of 2-(4-methoxyphenyl)acetaldehyde **11** (2.63 g, 17.5 mmol, 3.0 equiv.) dissolved in dry THF (9 mL), the reaction was allowed to warm to rt within 1 h. After the addition of potassium *tert*-butoxide (690 mg, 5.80 mmol, 1.0 equiv.), the reaction mixture was stirred for an additional hour at rt. The reaction was quenched with a saturated aqueous ammonium chloride solution (30 mL), and the aqueous layer was extracted with DCM (3 × 50 mL). The combined organic layers were washed with hydrochloric acid (2 M, 2 × 50 mL), and the aqueous layer was again extracted with DCM (3 × 50 mL). The combined organic layers were washed with brine (100 mL), dried with sodium sulfate, and filtered. The filtrate was concentrated in vacuo, and the crude residue was purified by silica gel column chromatography (EA/PE = 1:4) to give the product **22** (1.16 g, yield 85%) as a light-yellow oil. TLC: R_f_ = 0.26 (EA/PE = 1:4) [KMnO_4_]. ^1^H NMR (600 MHz, CDCl_3_) δ 7.08 (d, *J* = 8.6 Hz, 2H), 6.82 (d, *J* = 8.6 Hz, 2H), 5.83 (ddd, *J* = 16.6, 10.6, 5.6 Hz, 1H), 5.27 (d, *J* = 17.1 Hz, 1H), 5.14–5.08 (m, 1H), 4.61–4.52 (m, 1H), 3.77 (s, 3H), 2.88 (s, 1H), 2.84 (t, *J* = 7.6 Hz, 2H), 2.73 (t, *J* = 7.6 Hz, 2H), 2.60 (d, *J* = 6.4 Hz, 2H). ^13^C NMR (151 MHz, CDCl_3_) δ 210.2, 158.1, 139.2, 132.8, 129.3, 115.0, 114.0, 68.7, 55.3, 49.1, 45.5, 28.7. IR (ATR): $\tilde{\nu }$ [cm^−1^] = 2932, 1707, 1611, 1583, 1510, 1463, 1441, 1404, 1365, 1299, 1242, 1177, 1108, 1084, 1032, 976, 927, 823. [α]^20^_D_ = +11.3 (c = 1.21, DCM). HRMS (ESI) *m*/*z*: [M + Na^+^] calcd. for C_14_H_18_NaO_3_: 257.1148; found: 257.1148.

#### 3.2.11. (3*S*,5*R*)-5-Hydroxy-7-(4-methoxyphenyl)hept-1-en-3-yl acetate (**23a**)

To a solution of 5-hydroxy-1-(4-methoxyphenyl)hept-6-en-3-one **23a** (412 mg, 1.40 mmol, 1.0 equiv.) in dry THF (3 mL) was added acetaldehyde (347 µL, 6.15 mmol, 3.5 equiv.). At −50 °C, SmI_2_ (0.1 M in THF, 13.2 mL, 1.32 mmol, 0.80 equiv.) was added slowly under the exclusion of light. The reaction mixture was allowed to stir at −20 °C for 18 h. After the addition of a saturated aqueous sodium bicarbonate solution (15 mL), the layers were separated, and the aqueous layer was extracted with ethyl acetate (3 × 10 mL). The combined organic layers were washed with brine (20 mL), dried with sodium sulfate, and filtered. The filtrate was concentrated in vacuo, and the crude residue was purified by column chromatography (EA/CyHex = 1:4) to give the product **23a** (426 mg, yield 87%) as a colorless oil. TLC: R_f_ = 0.165 (EA/CyHex = 1:4) [KMnO_4_]. ^1^H NMR (600 MHz, CDCl_3_) δ 7.11 (d, *J* = 8.6 Hz, 2H), 6.82 (d, *J* = 8.6 Hz, 2H), 5.84 (ddd, *J* = 16.9, 10.5, 6.0 Hz, 1H), 5.52 (ddt, *J* = 9.5, 5.6, 3.6 Hz, 1H), 5.27 (dt, *J* = 17.3, 1.3 Hz, 1H), 5.16 (dt, *J* = 10.5, 1.3 Hz, 1H), 3.78 (s, 3H), 3.56 (tt, *J* = 7.8, 3.5 Hz, 1H), 2.75 (ddd, *J* = 14.8, 9.8, 5.5 Hz, 1H), 2.62 (ddd, *J* = 13.8, 9.7, 6.6 Hz, 2H), 2.08 (s, 3H), 1.84–1.63 (m, 4H). ^13^C NMR (151 MHz, CDCl_3_) δ 171.7, 158.1, 136.8, 129.6, 116.6, 72.4, 67.0, 55.6, 43.0, 39.4, 31.5, 21.4. IR (ATR): $\tilde{\nu }$ [cm^−1^] = 2928, 1608, 1510, 1463, 1247, 1174, 1037, 834, 774. [α]^20^_D_ = −3.1 (c = 1.0, DCM). HRMS (ESI) *m*/*z*: [M + Na^+^] calcd. for C_16_H_22_NaO_4_: 301.1410; found: 301.1410.

#### 3.2.12. (3*S*,5*R*)-7-(4-Methoxyphenyl)hept-1-ene-3,5-diol (**23**)

To a solution of (3*S*,5*R*)-5-hydroxy-7-(4-methoxyphenyl)hept-1-en-3-yl acetate **23a** (300 mg, 1.08 mmol, 1.0 equiv.) in MeOH/H_2_O (4 mL, 3:1) was added potassium carbonate (297 mg, 2.15 mmol, 2.0 equiv.) at 0 °C. The reaction mixture was stirred for 4 h at 0 °C and then allowed to warm up to room temperature. Upon completion of the reaction indicated by TLC analysis, H_2_O (10 mL) and ethyl acetate (10 mL) were added, the layers were separated, and the aqueous layer was extracted with ethyl acetate (3 × 10 mL). The combined organic layers were washed with brine (15 mL), dried with sodium sulfate, and filtered. The filtrate was concentrated in vacuo, and the crude residue was purified by column chromatography (EA/CyHex = 3:7) to give the product **23** (234 mg, yield 92%) as a colorless oil. TLC: R_f_ = 0.195 (EA/CyHex = 3:7) [KMnO_4_]. ^1^H NMR (600 MHz, CDCl_3_) δ 7.11 (d, *J* = 8.5 Hz, 2H), 6.83 (d, *J* = 8.6 Hz, 2H), 5.92 (ddd, *J* = 17.1, 10.5, 5.5 Hz, 1H), 5.28 (d, *J* = 17.3 Hz, 1H), 5.14 (d, *J* = 10.5 Hz, 1H), 4.51–4.44 (m, 1H), 4.00–3.93 (m, 1H), 3.78 (s, 3H), 2.73 (ddd, *J* = 13.8, 9.9, 5.7 Hz, 1H), 2.62 (ddd, *J* = 13.9, 9.7, 6.4 Hz, 1H), 2.25 (s, 2H), 1.87–1.61 (m, 4H). ^13^C NMR (151 MHz, CDCl_3_) δ 158.0, 140.8, 134.1, 129.4, 114.6, 114.0, 70.9, 68.9, 55.4, 42.5, 39.5, 31.3. IR (ATR): $\tilde{\nu }$ [cm^−1^] = 3354, 2935, 1611, 1510, 1242, 1177, 1034, 922, 822, 526. [α]^20^_D_ = +15.4 (c = 1.0, DCM). HRMS (ESI) *m*/*z*: [M + Na^+^] calcd. for C_14_H_20_NaO_3_: 259.1304; found: 259.1305.

#### 3.2.13. (5*R*,7*S*)-5-(4-Methoxyphenethyl)-2,2,3,3,9,9,10,10-octamethyl-7-vinyl-4,8-dioxa-3,9-disilaundecane (**24**)

To a dry round-bottom flask was added diol **23** (200 mg, 846 µmol, 1.0 equiv.) in DMF (5 mL), and the solution was cooled to 0 °C for 20 min. Imidazole (460 mg, 6.77 mmol, 8.0 equiv.) and *tert*-butyldimethylsilyl chloride (511 mg, 3.39 mmol, 4.0 equiv.) were added, and the solution was allowed to stir at rt for 18 h. Following the addition of distilled water (15 mL) and diethyl ether (15 mL), the layers were separated, and the aqueous layer was extracted with diethyl ether (3 × 15 mL). The combined organic layers were washed with brine (15 mL), dried with sodium sulfate, and filtered. The filtrate was concentrated in vacuo, and flash chromatography (EA/CyHex = 1:99) on silica gel afforded product **24** in 95% yield (374 mg, dr > 99:1) as a colorless oil. TLC: R_f_ = 0.195 (EA/CyHex = 3:7) [KMnO_4_]. ^1^H NMR (600 MHz, CDCl_3_) δ 7.10 (d, *J* = 8.4 Hz, 2H), 6.83 (d, *J* = 8.4 Hz, 2H), 5.81 (ddd, *J* = 17.2, 10.3, 7.0 Hz, 1H), 5.12 (d, *J* = 17.2 Hz, 1H), 5.03 (d, *J* = 10.3 Hz, 1H), 4.19 (d, *J* = 6.7 Hz, 1H), 3.87–3.80 (m, 1H), 3.79 (s, 3H), 2.60 (dddd, *J* = 38.9, 13.7, 10.6, 5.7 Hz, 1H), 1.82–1.69 (m, 3H), 1.65 (dt, *J* = 13.5, 6.0 Hz, 1H), 0.92 (s, 9H), 0.89 (s, 9H), 0.19–−0.17 (m, 12H). ^13^C NMR (151 MHz, CDCl_3_) δ 157.9, 142.2, 134.9, 129.4, 114.2, 72.1, 69.4, 55.4, 46.4, 40.0, 30.6, 26.1 (d, *J* = 5.0 Hz), 18.3, −3.7, −3.8, −4.0, −4.4. IR (ATR): $\tilde{\nu }$ [cm^−1^] = 2928, 1511, 1463, 1246, 1073, 833, 773. [α]^20^_D_ = +1.0 (c = 1.0, DCM). HRMS (ESI) *m*/*z*: [M + Na^+^] calcd. for C_26_H_48_O_3_NaSi_2_: 487.3034; found: 487.3034.

#### 3.2.14. (5*R*,7*S*)-5-(4-Methoxyphenethyl)-7-((*E*)-4-methoxystyryl)-2,2,3,3,9,9,10,10-octamethyl-4,8-dioxa-3,9-disilaundecane (**26**)

An oven-dried round-bottom flask was charged with (3*S*,5*R*)-7-(4-methoxyphenyl)hept-1-ene-3,5-diol **24** (320 mg, 688 µmol, 1.0 equiv.), palladium (II) acetate (46.4 mg, 210 µmol, 30 mol%), potassium carbonate (951 mg, 6.88 mmol, 10 equiv.), and tetra-*n*-butylammonium chloride (957 mg, 3.44 mmol, 5.0 equiv.). The flask was evacuated and backfilled with argon three times. 4-iodoanisole **25** (242 mg, 1.03 mmol, 1.5 equiv.) dissolved in DMF (5 mL; degassed by freeze–pump–thaw method) was added to the mixture. The mixture was allowed to stir at 90 °C for 19 h. After this time, the reaction mixture was allowed to cool down to room temperature and diluted with ethyl acetate (10 mL) and water (10 mL). The combined aqueous layers were extracted with ethyl acetate (3 × 10 mL), and the combined organic layers were washed with brine (8 mL), dried with sodium sulfate, and filtered. The filtrate was concentrated in vacuo, and flash chromatography (DE/PE = 1:50) on silica gel afforded the product **26** (300 mg, yield 84%, *dr* > 20:1) as a colorless oil. TLC: R_f_ = 0.195 (DE/PE = 1:50) [KMnO_4_]. ^1^H NMR (600 MHz, CDCl_3_) δ 7.29 (d, *J* = 8.6 Hz, 2H), 7.08 (d, *J* = 8.5 Hz, 2H), 6.86 (d, *J* = 8.7 Hz, 2H), 6.81 (d, *J* = 8.5 Hz, 2H), 6.37 (d, J = 15.9 Hz, 1H), 6.00 (dd, *J* = 15.8, 7.5 Hz, 1H), 4.34 (q, *J* = 6.9 Hz, 1H), 3.87 (p, *J* = 5.8 Hz, 1H), 3.82 (s, 3H), 3.78 (s, 3H), 2.60 (tq, J = 19.6, 6.8 Hz, 2H), 1.89–1.68 (m, 4H), 0.92 (s, 9H), 0.90 (s, 9H), 0.09–0.02 (m, 12H). ^13^C NMR (151 MHz, CDCl_3_) δ 159.3, 157.8, 134.8, 131.5, 130.0, 129.4, 129.1, 127.7, 114.2, 113.9, 71.8, 69.3, 55.4 (d, *J* = 7.6 Hz), 46.7, 40.1, 30.6, 18.3, −3.5, −3.9, −4.0, −4.3.z IR (ATR): $\tilde{\nu }$ [cm^−1^] = 2929, 1607, 1502, 1275, 1250, 1182, 1040, 906, 823, 809, 731. [α]^20^_D_ −23.0 (c = 1.0, DCM). HRMS (ESI) *m*/*z*: [M + Na^+^] calcd. for C_33_H_54_O_4_NaSi_2_: 593.3561; found: 593.3452.

#### 3.2.15. (5*R*,7*R*)-5,7-bis(4-Methoxyphenethyl)-2,2,3,3,9,9,10,10-octamethyl-4,8-dioxa-3,9-disilaundecane (**27**)

In an oven-dried round-bottom flask, (5*R*,7*S*)-5-(4-methoxyphenethyl)-7-((*E*)-4-methoxystyryl)-2,2,3,3,9,9,10,10-octamethyl-4,8-dioxa-3,9-disilaundecane **26** (200 mg, 350 µmol, 1.0 equiv.) was dissolved in methanol (8 mL), and Pd/C (10% on activated charcoal, 112 mg, 105 µmol, 30 mol%) was added. The reaction mixture was stirred for 8 h at room temperature under a hydrogen atmosphere (1 atm). The suspension was then filtered through a pad of celite and washed (3 × 10 mL) with ethyl acetate. The filtrate was concentrated in vacuo, and flash chromatography (EA/CyHex = 1:9) on silica gel afforded product **27** (193 mg, yield 93%) as a colorless oil. TLC: R_f_ = 0.21 (EA/CyHex = 1:17) [KMnO_4_]. ^1^H NMR (600 MHz, CDCl_3_) δ 7.09 (d, *J* = 8.1 Hz, 4), 6.83 (d, *J* = 8.6 Hz, 4H), 3.80 (d, *J* = 6.2 Hz, 8H), 2.67–2.53 (m, 4H), 1.72 (dt, *J* = 12.1, 5.8 Hz, 6H), 0.91 (s, 18H), 0.07 (d, *J* = 6.8 Hz, 12H). ^13^C NMR (151 MHz, CDCl_3_) δ 157.9, 134.8, 129.4, 114.0, 69.9, 55.4, 45.4, 40.1, 30.6, 26.1, 18.3, −3.9. IR (ATR): $\tilde{\nu }$ [cm^−1^] = 2928, 1511, 1462, 1244, 1176, 1037, 832, 771. [α]^20^_D_ = +2.1 (c = 1.0, DCM). HRMS (ESI) *m*/*z*: [M + Na^+^] calcd. for C_33_H_56_O_4_NaSi_2_: 595.3717; found: 595.3608.

#### 3.2.16. (3*R*,5*R*)-1,7-bis(4-Methoxyphenyl)heptane-3,5-diol (**4**)

First, HF-pyridine (70% HF, 250 µL, 1.75 mmol, 20 equiv.) was dissolved in a pyridine/MeOH mixture (890 µL, 6:1) at 0 °C in a Teflon vial. In another Teflon vial, (5*R*,7*R*)-5,7-bis(4-methoxyphenethyl)-2,2,3,3,9,9,10,10-octamethyl-4,8-dioxa-3,9-disilaundecane **27** (50.0 mg, 871 μmol, 1.0 equiv.) was dissolved in THF (0.8 mL), and the solution was cooled to 0 °C. The prepared HF-pyridine solution was then transferred to the THF solution, and the reaction mixture was stirred at rt for 18 h. Following the addition of an excess of methoxytrimethylsilane (7 mL) at 0 °C and dilution with toluene (10 mL), the volatile compounds were removed in vacuo. The residue was diluted with toluene (5 mL) again, and the procedure was repeated three times to remove all of the pyridine. The crude residue was purified by silica gel column chromatography (EA/CyHex = 2:5) to give 1,3 diol **4** (26.2 mg, yield 87%) as a white solid. ^1^H NMR (600 MHz, CDCl_3_) δ 7.11 (d, *J* = 8.4 Hz, 4H), 6.83 (d, *J* = 8.6 Hz, 4H), 3.97 (dd, *J* = 8.4, 5.1 Hz, 2H), 3.78 (s, 6H), 2.71 (ddd, *J* = 15.0, 9.7, 5.7 Hz, 2H), 2.61 (ddd, *J* = 13.8, 9.5, 6.5 Hz, 2H), 2.12 (s, 2H), 1.83 (ddd, *J* = 14.3, 8.7, 5.6 Hz, 2H), 1.80 (d, *J* = 5.7 Hz, 2H), 1.73 (dddd, *J* = 13.9, 10.4, 6.5, 4.6 Hz, 2H), 1.66 (t, *J* = 5.6 Hz, 2H). ^13^C NMR (151 MHz, CDCl_3_) δ 158.0, 134.0, 129.4, 114.1, 69.1, 55.4, 42.7, 39.4, 31.4. IR (ATR): $\tilde{\nu }$ [cm^−1^] = 3374, 2939, 1612, 1511, 1300, 1244, 1176, 1033, 814, 514. [α]^20^_D_ = +6.5 (c = 1.0, DCM). HRMS (ESI) *m*/*z*: [M + Na^+^] calcd. for C_21_H_28_NaO_4_: 367.1988; found: 367.1879. All the characterization data are consistent with the literature [11].

## 4. Conclusions

In summary, we successfully completed the formal synthesis of (+)-hannokinol **1** by employing a Horner–Wittig reaction and Pd-catalyzed Heck cross coupling as key steps. The key stereochemical information for the 1,3-diol unit was introduced by the use of chiral building block **7** (prepared from readily available and inexpensive 2-deoxy-D-ribose) and a directed *anti*-reduction under Evans–Tishchenko conditions. Our approach enables the synthesis of precursor **4** in high stereoselectivity and yields (eight steps, 37%, excluding the synthesis of **7**).

## Data Availability

All experimental data supporting our findings can be found in the Supplementary Materials.

## Supplementary Materials

The following supporting information can be downloaded at: https://www.mdpi.com/article/10.3390/molecules29153710/s1, Experimental procedures and compound characterization. References [16,18,36,37,38] are cited in the Supplementary Materials.
