# Supplementary material for: A Formal Synthesis of (+)-Hannokinol Using a Chiral Horner–Wittig Reagent
Source: Molecules. 2024 Aug 5;29(15):3710. doi: 10.3390/molecules29153710 (PMC11313872; doi:10.3390/molecules29153710)
Supplement: Supplementary file 1 [file molecules-29-03710-s001.zip › molecules-3100642-supplementary.pdf]

<u>**Supporting Information**</u>

A Formal Synthesis of (+)-Hannokinol Using a Chiral Horner–Wittig Reagent

Michael Tapera, Federica Borghi, Jan Lukas Mayer-Figge, Fabia Mittendorf, Ibrahim-Ethem Celik, Adrián Gómez-Suárez and Stefan F. Kirsch *

Organic Chemistry, Bergische Universität Wuppertal, Gaußstr. 20, 42119 Wuppertal, Germany
* Correspondence: sfkirsch@uni-wuppertal.de

Contents

Synthesis of Chiral Building Block 3
References 6
Spectra 7
^{1}H, ^{13}C and HRMS spectra of methyl 2-(4-(benzyloxy)phenyl)acetate (**13**) 7
^{1}H, and ^{13}C spectra of 2-(4-(benzyloxy)phenyl)ethan-1-ol (**14**) 9
^{1}H, ^{13}C and HRMS spectra of 2-(4-(benzyloxy)phenyl)acetaldehyde (**11a**) 10
^{1}H, ^{13}C and HRMS spectra of (S)-1-(4-(benzyloxy)phenyl)-5-hydroxyhept-6-en-3-one (**15**) 12
^{1}H, ^{13}C and HRMS spectra of (3S,5R)-7-(4-(benzyloxy)phenyl)hept-1-ene-3,5-diol (**16**) 14
^{1}H, ^{13}C and HRMS spectra of ((((3S,5R)-7-(4-(benzyloxy)phenyl)hept-1-ene-3,5-diyl)bis(oxy))bis(methylene))dibenzene (**17**) 16
^{1}H, ^{13}C and HRMS spectra of 4,4'-((3S,5R,E)-3,5-bis(benzyloxy)hept-1-ene-1,7-diyl)bis((benzyloxy)benzene) (**19**) 18
^{1}H, ^{13}C and HRMS spectra of methyl 2-(4-methoxyphenyl)acetate (**21**) 20
^{1}H, ^{13}C and HRMS spectra of 2-(4-methoxyphenyl)acetaldehyde (**11**) 22
^{1}H, ^{13}C and HRMS spectra of 5-hydroxy-1-(4-methoxyphenyl)hept-6-en-3-one (**22**) 24
^{1}H, ^{13}C and HRMS spectra of (3S,5R)-5-hydroxy-7-(4-methoxyphenyl)hept-1-en-3-yl acetate (**23a**) 26
^{1}H, ^{13}C and HRMS spectra of (3S,5R)-7-(4-methoxyphenyl)hept-1-ene-3,5-diol (**23**) 28
^{1}H, ^{13}C and HRMS spectra of (5R,7S)-5-(4-methoxyphenethyl)-2,2,3,3,9,9,10,10-octamethyl-7-vinyl-4,8-dioxa-3,9-disilaundecane (**24**) 30
^{1}H, ^{13}C and HRMS spectra of (5R,7S)-5-(4-methoxyphenethyl)-7-((E)-4-methoxystyryl)-2,2,3,3,9,9,10,10-octamethyl-4,8-dioxa-3,9-disilaundecane (**26**) 32
^{1}H and ^{13}C spectra of (5R,7R)-5,7-bis(4-Methoxyphenethyl)-2,2,3,3,9,9,10,10-octamethyl-4,8-dioxa-3,9-disilaundecane (**27**) 34
^{1}H and ^{13}C spectra of (3R,5R)-1,7-bis(4-methoxyphenyl)heptane-3,5-diol (**4**) 36

Synthesis of Chiral Building Block

The chiral building block was prepared as described by Kirsch and coworkers [1].

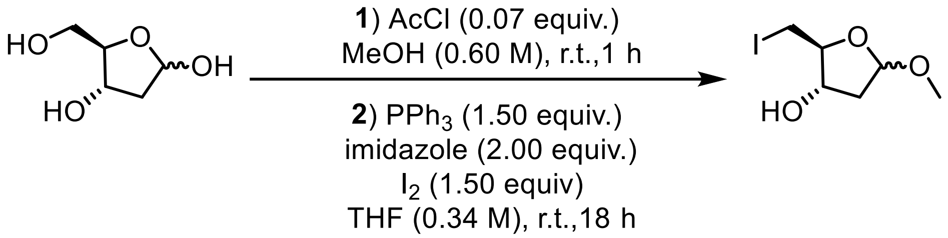

Acetyl chloride (371 μL, 3.09 mmol, 0.07 equiv.) was added to a solution of 2-deoxy-D-ribose (10.0 g, 74.6 mmol, 1.0 equiv.) in methanol (125 mL). After the reaction mixture was stirred at room temperature for 1 h, sodium bicarbonate (4.38 g, 52.2 mmol, 0.70 equiv.) was added and the suspension was stirred for 5 mins. The reaction mixture was then filtered through a pad of Celite; the residue was washed several times with methanol (3 × 10 mL). The filtrate was concentrated in vacuo at a water bath temperature of 40 °C, and crude acetal (11.0 g, 75.3 mmol) was dissolved in THF (220 mL). Triphenylphosphine (29.2 g,111 mmol, 1.5 equiv.), imidazole (10.1 g, 148 mmol, 2.00 equiv.), and iodine (28.3 g, 111 mmol, 1.50 equiv.) were added successively, and the suspension was stirred at room temperature for 18 h and then filtered through a pad of celite; the residue was washed several times with ethyl acetate (3 ×50 mL). The filtrate was concentrated in vacuo, and flash chromatography (CyH/EtOAc 7:3) on silica gel afforded product in 90% yield (17.4 g, 67.4 mmol, *dr* 67:33) as a colorless oil. TLC: Rf= 0.19 (CyH/EtOAc = 7:3) [KMnO4]. ^{1}H NMR (600 MHz, $CDCl_3$):δ 5.13 (d, J = 4.7 Hz, 1H), 5.10 (dd, J = 5.4, 1.8 Hz, 1H), 4.46 (ddd, J= 10.7, 6.7, 4.1 Hz, 1H), 4.14–4.06 (m, 3H), 3.38 (s, 3H), 3.35 (s,3H), 3.30 (dd, J = 9.8, 5.7 Hz, 1H), 3.25 (dd, J = 10.5, 4.9 Hz, 1H),3.21–3.16 (m, 2H), 2.31 (ddd, J = 13.6, 6.9, 1.7 Hz, 1H), 2.23 (ddd, J= 14.0, 6.6, 4.7 Hz, 1H), 2.12 (ddd, J = 13.6, 6.2, 5.4 Hz, 1H), 2.00(dd, J = 14.0, 1.3 Hz, 1H). ^{13}C{^{1}H} NMR (151 MHz, $CDCl_3$): δ105.9, 105.5, 86.2, 86.1, 75.8, 75.6, 55.4, 55.1, 42.0, 41.0, 7.9, 6.7.

The analytical data are in agreement with previously reported data [2].

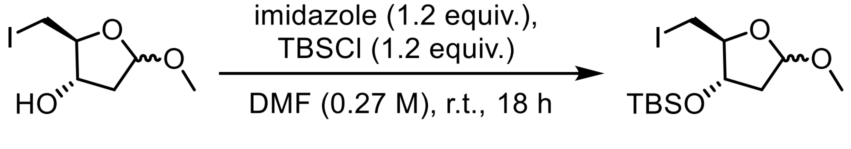

The iodide (16.7 g, 64.8 mmol, 1.00 equiv.) was dissolved in DMF (236 mL), and the solution was cooled to 0 °C. Imidazole (5.29 g, 77.7 mmol, 1.20 equiv.) and tert-butyl dimethylsilylchloride (11.7 g, 77.7 mmol, 1.20 equiv.) dissolved in DCM (78 mL) were added. The reaction mixture was stirred at room temperature for 18 h and then diluted with heptane (100 mL). The organic layer was washed with H_2O (3 × 50 mL) and brine (50 mL), dried with sodium sulfate, and filtered. The filtrate was concentrated in vacuo, and flash chromatography (CyH/EtOAc 9:1) on silica gel afforded product in 95% yield (22.9 g, 61.4 mmol, *dr* 54:46) as a colorless oil. TLC: Rf= 0.59 (CyH/EtOAc = 9:1) [$KMnO_4$, UV]. ^{1}H NMR (600 MHz,$CDCl_3$): δ 5.10 (dd, J = 5.4, 1.9 Hz, 0.5H), 5.00 (dd, J = 5.9, 3.0 Hz,0.5H), 4.43–4.29 (m, 0.5H), 4.00–3.87 (m, 1H), 3.60–3.54 (m,0.5H), 3.47 (dd, J = 10.8, 3.6 Hz, 0.5H), 3.39 (s, 1.5H), 3.37 (s,1.5H), 3.33–3.18 (m, 1.5H), 2.48 (ddd, J = 13.6, 8.6, 5.9 Hz, 0.5H),2.21 (ddd, J = 13.2, 6.6, 1.9 Hz, 0.5H), 2.06 (ddd, J = 13.2, 6.2, 5.4Hz, 0.5H), 1.84 (ddd, J = 13.6, 6.0, 3.0 Hz, 0.5H), 0.88 (s, 9H), 0.11(s, 1.5H), 0.08 (d, J = 0.9 Hz, 3H), 0.07 (s, 1.5H). ^{13}C{^{1}H} NMR(151 MHz, CDCl3): δ 105.4, 104.2, 86.0, 80.8, 75.7, 75.4, 55.42,55.40, 42.6, 42.1, 25.90, 25.86, 18.1, 18.0, 8.0, 7.9, −4.35, −4.36,−4.46, −4.51.

The analytical data are in agreement with previously reported data [3].

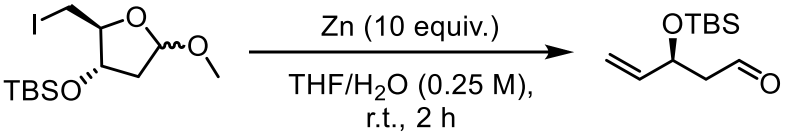

Activated zinc powder (stirring for 15 min in 1 M HCl, 24.6 g, 376 mmol, 10 equiv.) was added to a solution of silyl ether 10 (14.0 g, 37.6 mmol, 1.0 equiv.) in THF/H_2O (150 mL, 4:1). The reaction mixture was stirred at 90 °C for 1.5 h and then filtered through a pad of Celite. The residue was washed with diethyl ether (3 × 50 mL) and the filtrate was diluted with water (100 mL). The layers were separated, and the aqueous layer was extracted with diethyl ether (3 × 50 mL). The combined organic layers were washed with brine (50 mL), dried with sodium sulfate, and filtered. The filtrate was concentrated in vacuo and flash chromatography (CyH/EtOAc 9:1) on silica gel afforded product in 98% yield (7.90 g, 37.0 mmol) as a colorless oil. TLC: Rf= 0.58 (CyH/EtOAc = 9:1) [$KMnO_4$]. ^{1}H NMR (400 MHz, $CDCl_3$):δ 9.77 (dd, J = 2.7, 2.2 Hz, 1H), 5.87 (ddd, J = 17.1, 10.4, 5.8 Hz,1H), 5.26 (dt, J = 17.2, 1.5 Hz, 1H), 5.12 (dt, J = 10.4, 1.4 Hz, 1H),4.70–4.60 (m, 1H), 2.60 (ddd, J = 15.7, 6.8, 2.7 Hz, 1H), 2.52 (ddd, J= 15.7, 5.0, 2.2 Hz, 1H), 0.88 (s, 9H), 0.07 (s, 6H), 0.05 (s, 3H). ^{13}C{^{1}H} NMR (101 MHz, $CDCl_3$): δ 201.7, 140.1, 115.0, 69.6, 51.4,25.9, 18.2, −4.2, −4.9.

The analytical data are in agreement with previously reported data [4].

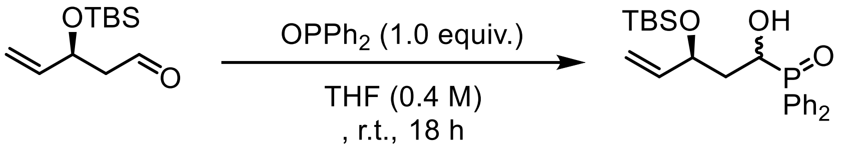

Diphenylphosphine oxide (4.94 g, 24.4 mmol, 1.0 equiv.) was added to a solution of aldehyde (5.40 g, 25.2 mmol, 1.0 equiv.) in dry THF (63 mL). The reaction mixture was stirred at 90°C for 18 h. The volatile compounds were evaporated under reduced pressure, and flash chromatography (DCM/EtOAc 4:6) on silica gel afforded product in 96% yield (10.1 g, 24.3 mmol, *dr* 60:40) as acolorless solid. TLC: Rf = 0.31 (DCM/EtOAc = 4:6) [$KMnO_4$, UV].^{1}H NMR (600 MHz, $CDCl_3$): δ 8.01–7.65 (m, 4H), 7.60–7.38 (m,6H), 5.84 (ddd, J = 17.1, 10.5, 4.9 Hz, 0.6H), 5.75 (ddd, J = 17.2,10.3, 6.9 Hz, 0.4H), 5.27 (dt, J = 17.2, 1.6 Hz, 0.6H), 5.22–5.12 (m,1H), 5.06 (dt, J = 10.4, 1.2 Hz, 0.4H), 4.82 (dt, J = 11.7, 2.1 Hz,0.6H), 4.68 (ddd, J = 11.0, 3.8, 1.5 Hz, 0.4H), 4.63–4.55 (m, 0.6H),4.52–4.43 (m, 0.4H), 2.18–2.10 (m, 0.4H), 2.03–1.96 (m, 0.6H),1.92–1.83 (m, 0.6H), 1.79–1.68 (m, 0.4H), 0.87 (s, 3H), 0.86 (s,6H), 0.08 (s, 1H), 0.04 (s, 1H), 0.02 (s, 2H), 0.02 (s, 2H). ^{13}C{^{1}H}NMR (151 MHz, $CDCl_3$): δ 140.7, 139.0, 132.45, 132.43, 132.39,132.37, 132.1, 132.0, 131.8, 131.73, 131.70, 131.66, 128.70, 128.69,128.62, 128.61, 128.5, 128.4, 115.53, 115.47, 75.7, 75.69, 72.59, 72.5,70.5, 69.9, 68.5, 67.9, 37.8, 36.3, 26.0, 25.9, 18.21, 18.16, −3.9, −4.5,−4.6, −5.3.

The analytical data are in agreement with previously reported data [5].

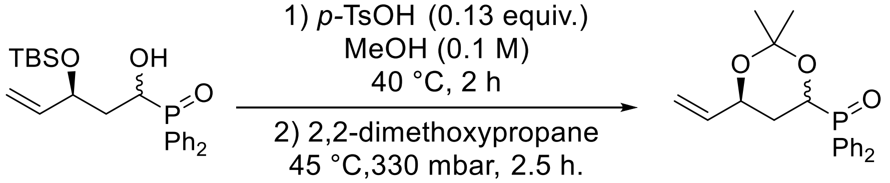

7

To a solution of alcohol (10 g, 24.0 mmol, 1.0 equiv.) in methanol (240 mL) was added *p*-toluenesulfonic acid monohydrate (594 mg, 3.12 mmol, 0.13 equiv.). The solution was stirred at 40 °C for 2.5 h. The solvent was removed in vacuo, and the residue was dissolved in 2,2-dimethoxypropane (75.3 mL, 63.8 g, 600 mmol, 25 equiv.). The reaction mixture was rotated at 45 °C for 2.5 h at 330 mbar in a rotavap and then diluted with dichloromethane (100 mL). The mixture was washed with saturated aqueous sodium bicarbonate solution (50 mL), dried with sodium sulfate, and filtered. The filtrate was concentrated in vacuo, and flash chromatography (DCM/EtOAc 4:6) on silica gel afforded product in 96% yield (7.88 g, 23.0 mmol, *dr* 70:30)

as a colorless solid. TLC: Rf = 0.64(DCM/EtOAc = 4:6) [$KMnO_4$, UV]. ^{1}H NMR (600 MHz, C_6D_6): δ8.25−7.93 (m, 4H), 7.15−7.06 (m, 6H), 5.67 (ddd, J = 17.3, 10.6, 5.3Hz, 0.3H), 5.58 (ddd, J = 17.3, 10.6, 5.1 Hz, 0.7H), 5.13−4.99 (m,1H), 4.93−4.81 (m, 1H), 4.78−4.65 (m, 1H), 4.26−4.15 (m, 0.3H),4.15−4.01 (m, 0.7H), 2.42−2.30 (m, 0.3H), 2.12−2.04 (m, 0.7H),2.03−1.93 (m, 0.3H), 1.76−1.62 (m, 0.7H), 1.37 (s, 2H), 1.24 (s,1H), 1.22 (s, 1H), 1.14 (s, 2H). ^{13}C{^{1}H} NMR (151 MHz, C_6D_6): δ138.6, 138.4, 132.92, 132.87, 132.8, 132.7, 131.89, 131.87, 131.82,131.80, 131.75, 131.72, 128.7, 128.65, 128.61, 128.5, 128.44, 128.40,128.37, 128.32, 114.8, 114.5, 101.5, 101.4, 99.53, 99.46, 70.2, 70.1,69.7, 69.1, 67.35, 67.75, 66.4, 65.7, 31.1, 30.2, 30.0, 25.1, 25.0, 19.1.

The analytical data are in agreement with previously reported data [5].

References

1. Mittendorf, F.; Celik, I.-E.; Kirsch, S.F. Total Synthesis of Cryptoconcatone D via Construction of 1,3-Diol Units Using Chiral Horner-Wittig Reagents. *J. Org. Chem.* **2022**, *87*, 14899–14908.
2. Dangerfield, E.M.; Plunkett, C.H.; Stocker, B.L.; Timmer, M.S.M. Protecting-group-free synthesis of 2-deoxy-aza-sugars. *Molecules* **2009**, *14*, 5298–5307.
3. Wang, D.; Nugent, W.A. 2-deoxyribose as a rich source of chiral 5-carbon building blocks. *J. Org. Chem.* **2007**, *72*, 7307–7312.
4. Tan, C.-H.; Holmes, A.B. The Synthesis of (+)-Allopumiliotoxin 323B′. *Chemistry* **2001**, *7*, 1845–1854.
5. Bredenkamp, A.; Wegener, M.; Hummel, S.; Häring, A.P.; Kirsch, S.F. Versatile process for the stereodiverse construction of 1,3-polyols: iterative chain elongation with chiral building blocks. *Chem. Commun. (Camb)* **2016**, *52*, 1875–1878.

Spectra

^{1}H, ^{13}C and HRMS spectra of methyl 2-(4-(benzyloxy)phenyl)acetate (**13**)

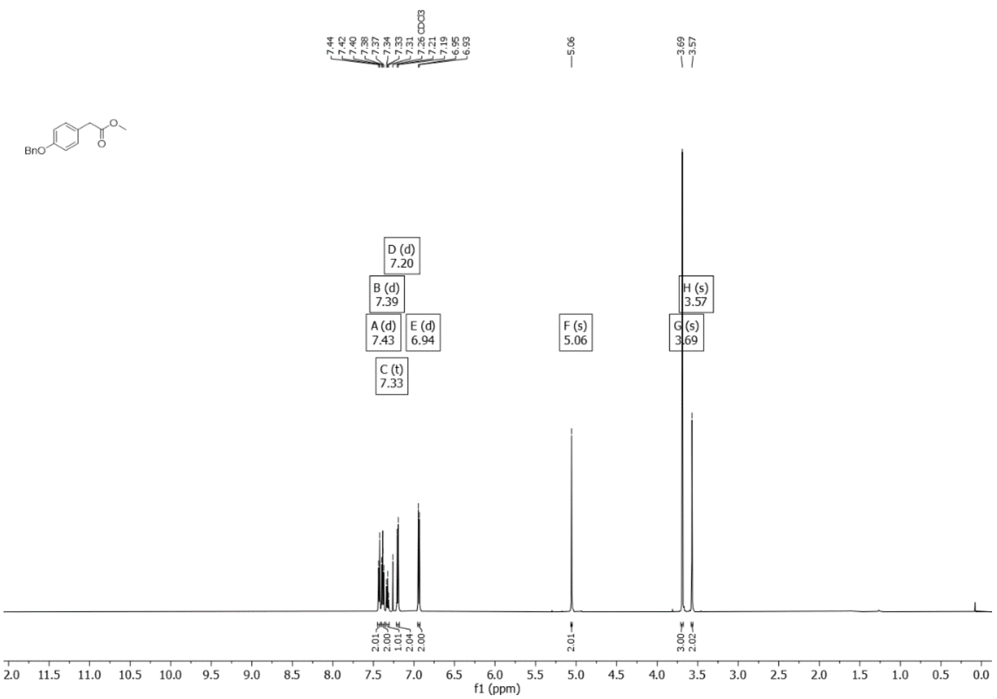

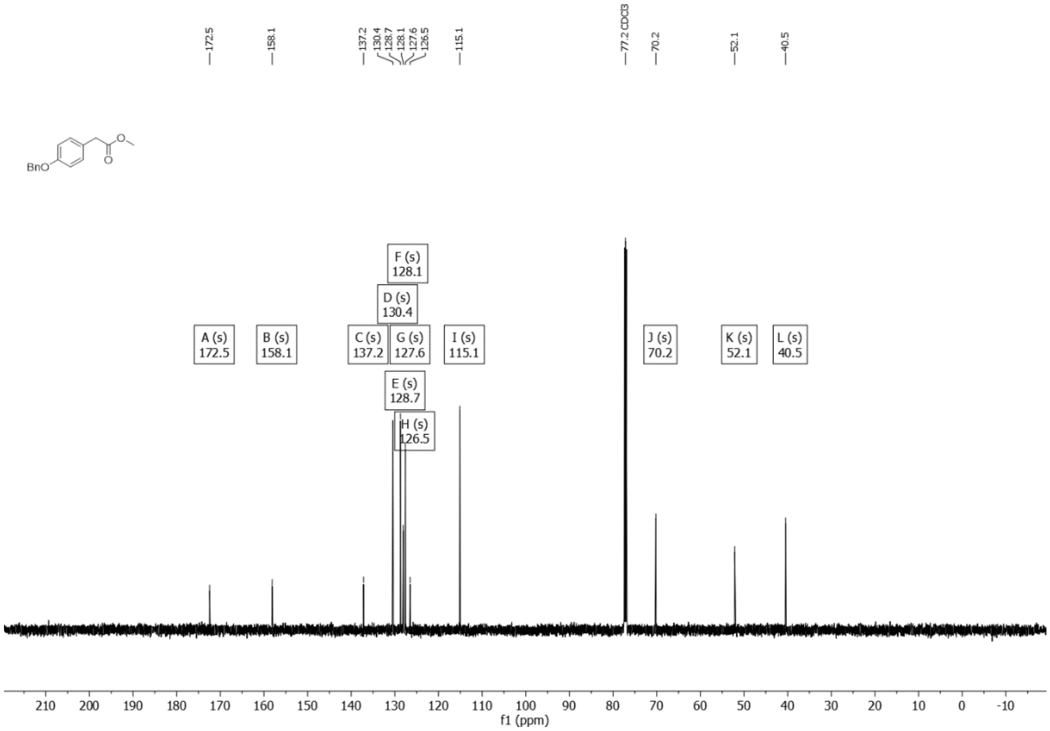

Acquisition Parameter

Source Type	ESI	Ion Polarity	Positive	Set Nebulizer	0.4 Bar
Focus	Not active			Set Dry Heater	200 °C
Scan Begin	50 m/z	Set Capillary	4500 V	Set Dry Gas	4.0 l/min
Scan End	1000 m/z	Set End Plate Offset	-500 V	Set Divert Valve	Waste

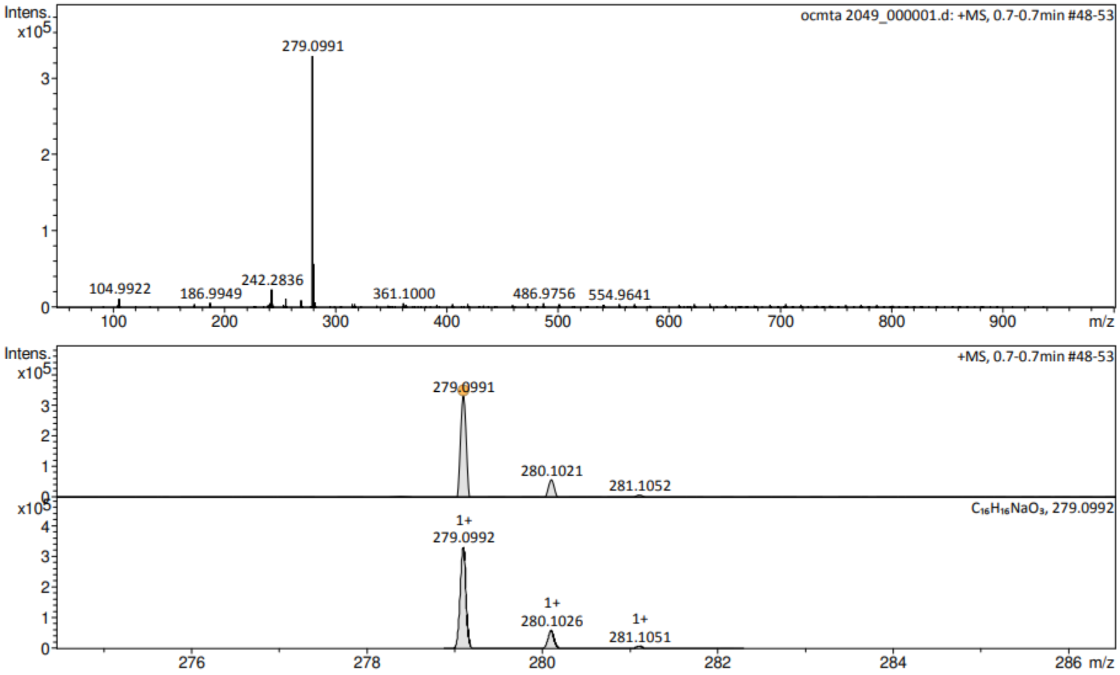

Meas. m/z	#	Ion Formula	m/z	err [ppm]	mSigma	# mSigma	Score	rdb	e⁻ Conf	N-Rule
279.0991	1	C16H16NaO3	279.0992	0.1	2.1	1	100.00	8.5	even	ok

^{1}H, and ^{13}C spectra of 2-(4-(benzyloxy)phenyl)ethan-1-ol (**14**)

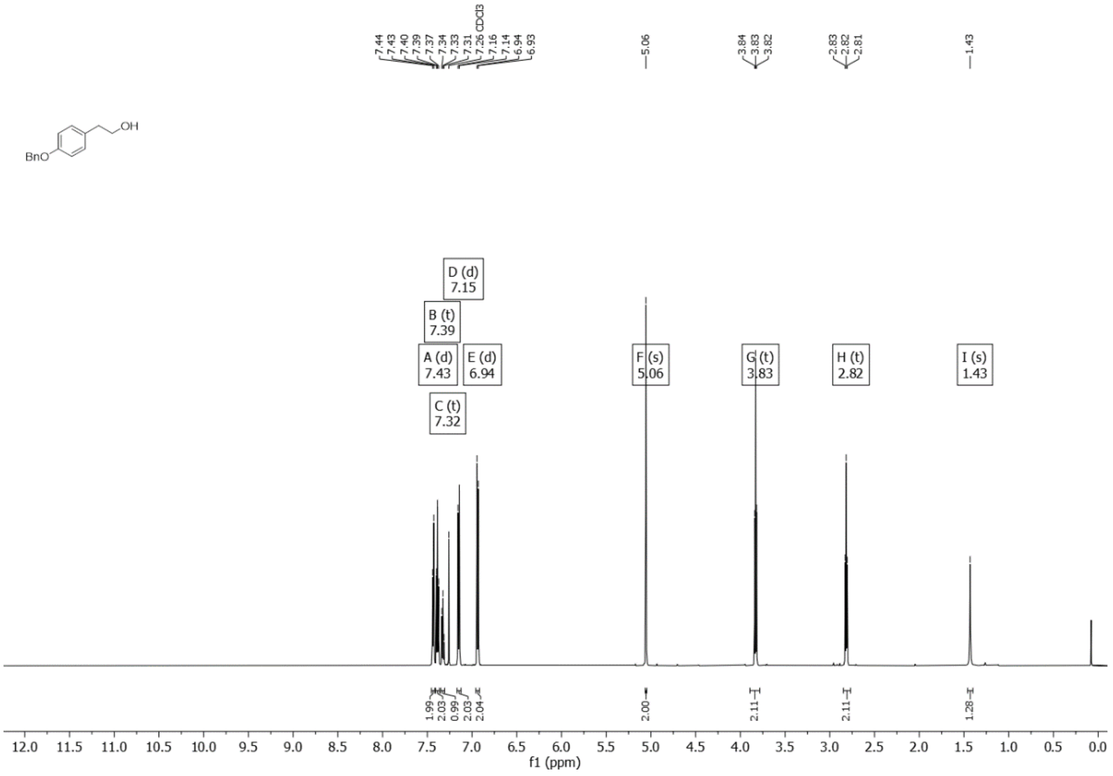

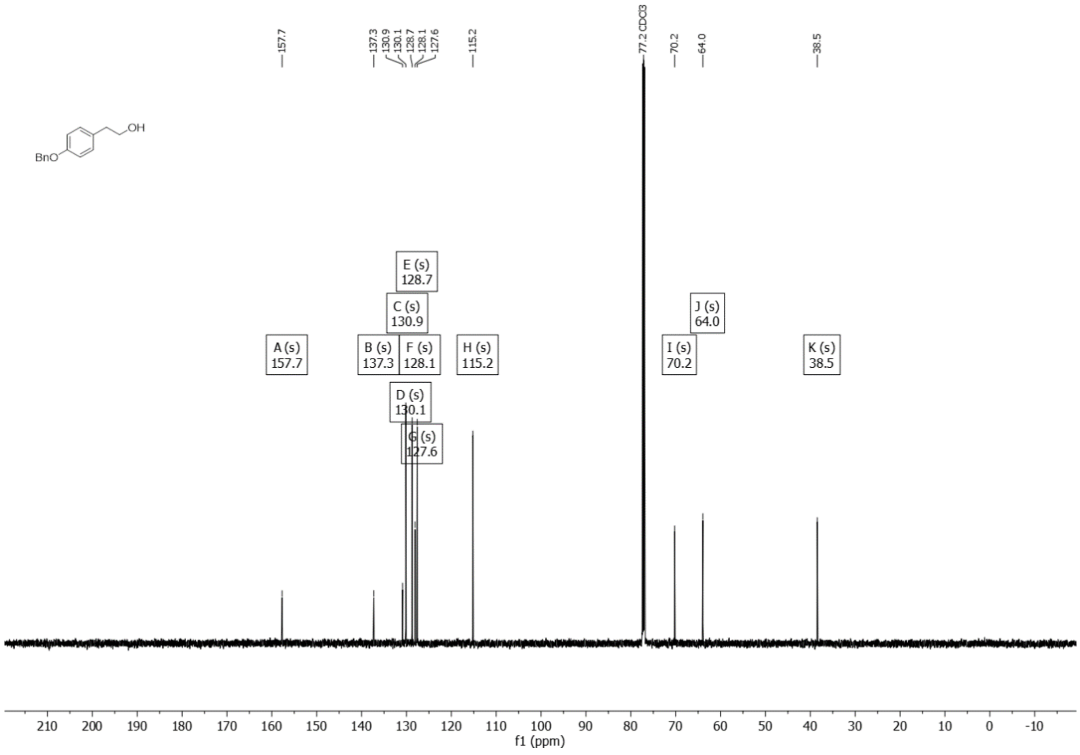

^{1}H, ^{13}C and HRMS spectra of 2-(4-(benzyloxy)phenyl)acetaldehyde (**11a**)

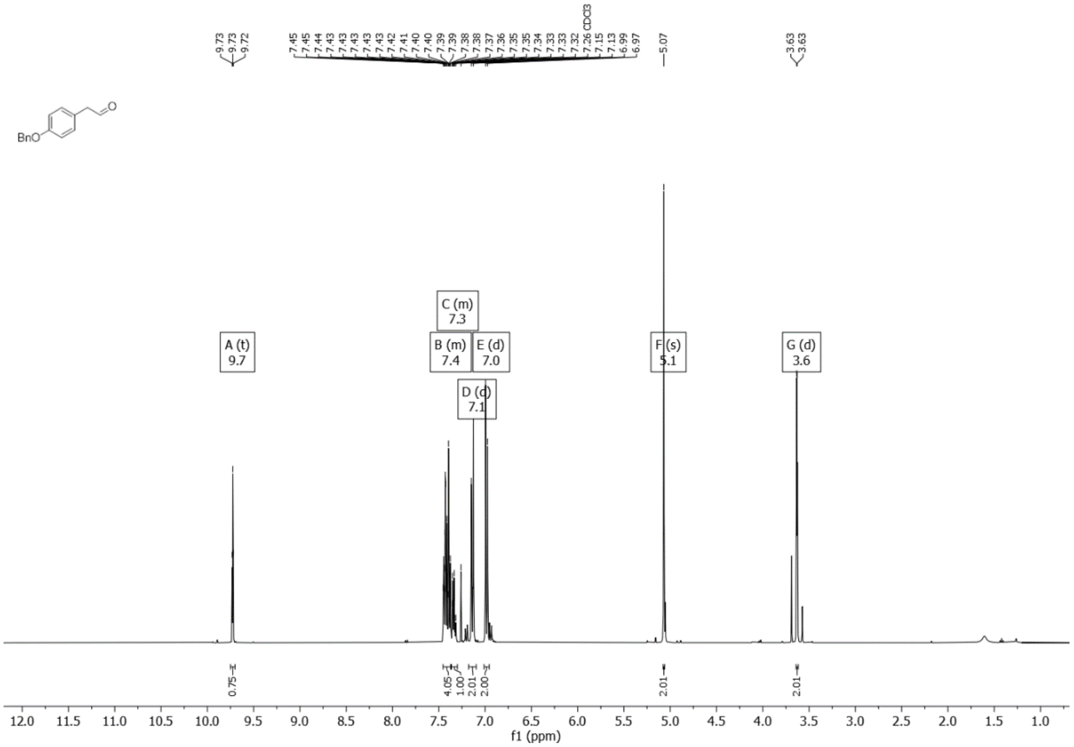

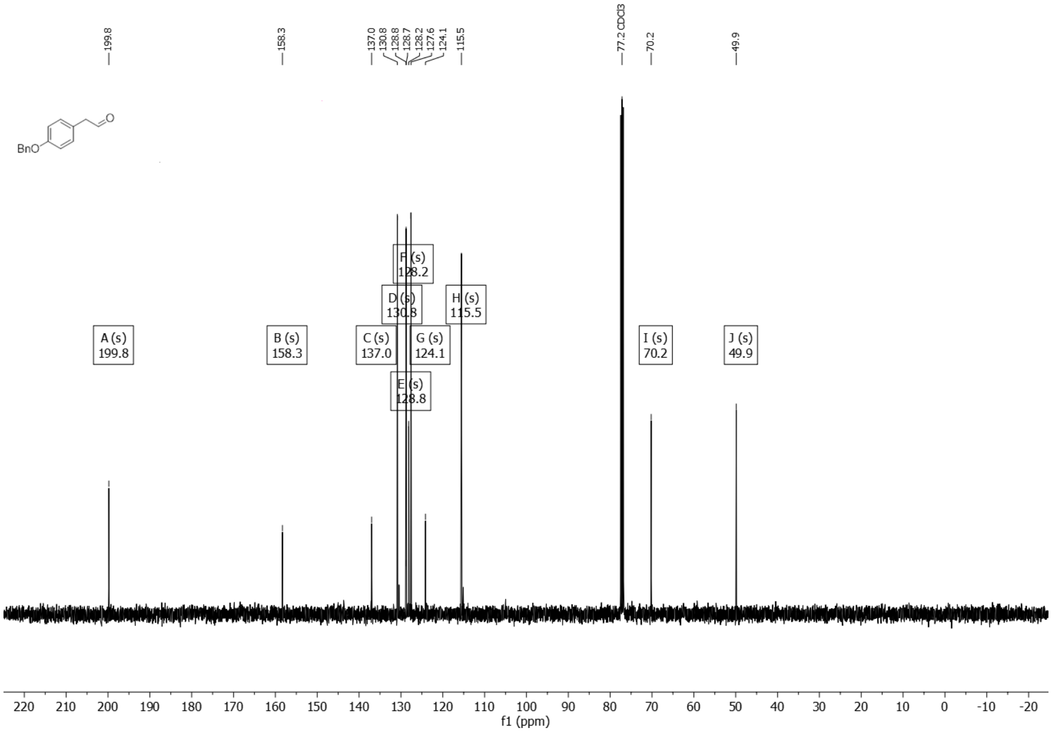

Acquisition Parameter

Source Type	ESI	Ion Polarity	Positive	Set Nebulizer	0.4 Bar
Focus	Not active			Set Dry Heater	200 °C
Scan Begin	50 m/z	Set Capillary	4500 V	Set Dry Gas	4.0 l/min
Scan End	1000 m/z	Set End Plate Offset	-500 V	Set Divert Valve	Waste

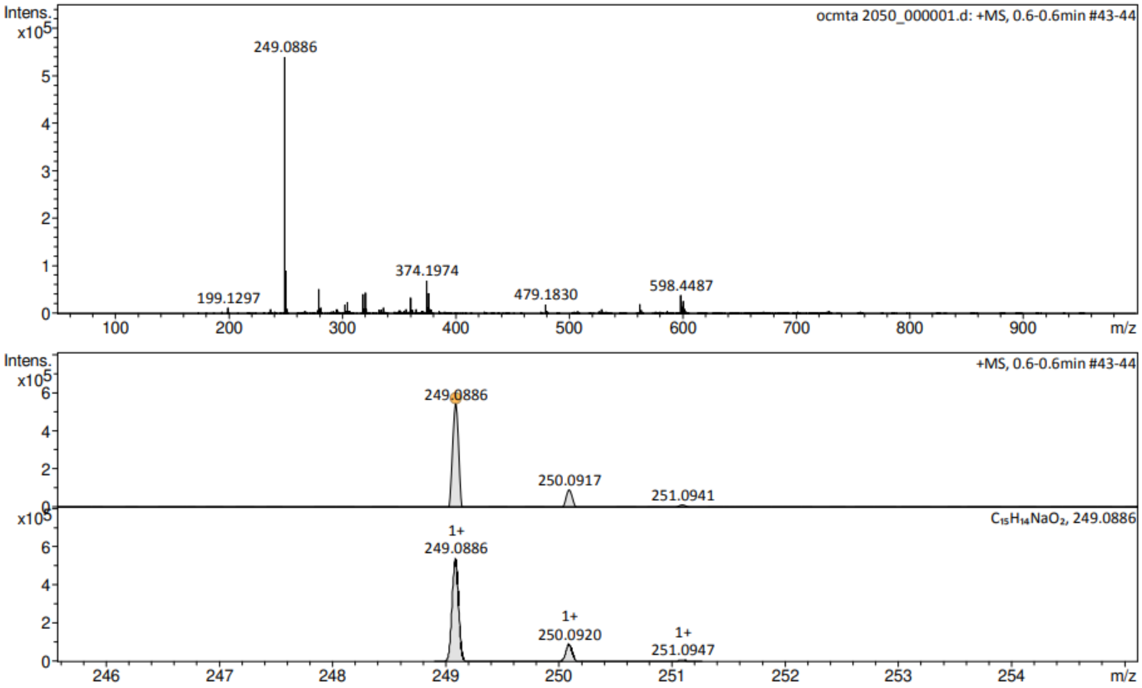

Meas. m/z	#	Ion Formula	m/z	err [ppm]	mSigma	# mSigma	Score	rdb	e⁻ Conf	N-Rule
249.0886	1	C15H14NaO2	249.0886	0.2	1.0	1	100.00	8.5	even	ok

^{1}H, ^{13}C and HRMS spectra of (S)-1-(4-(benzyloxy)phenyl)-5-hydroxyhept-6-en-3-one (**15**)

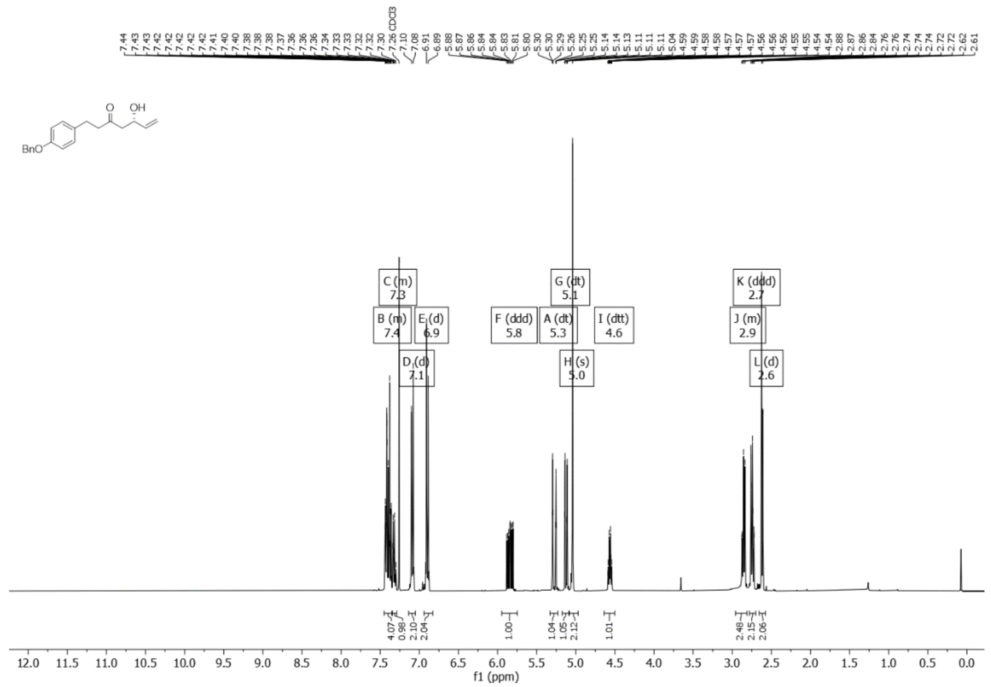

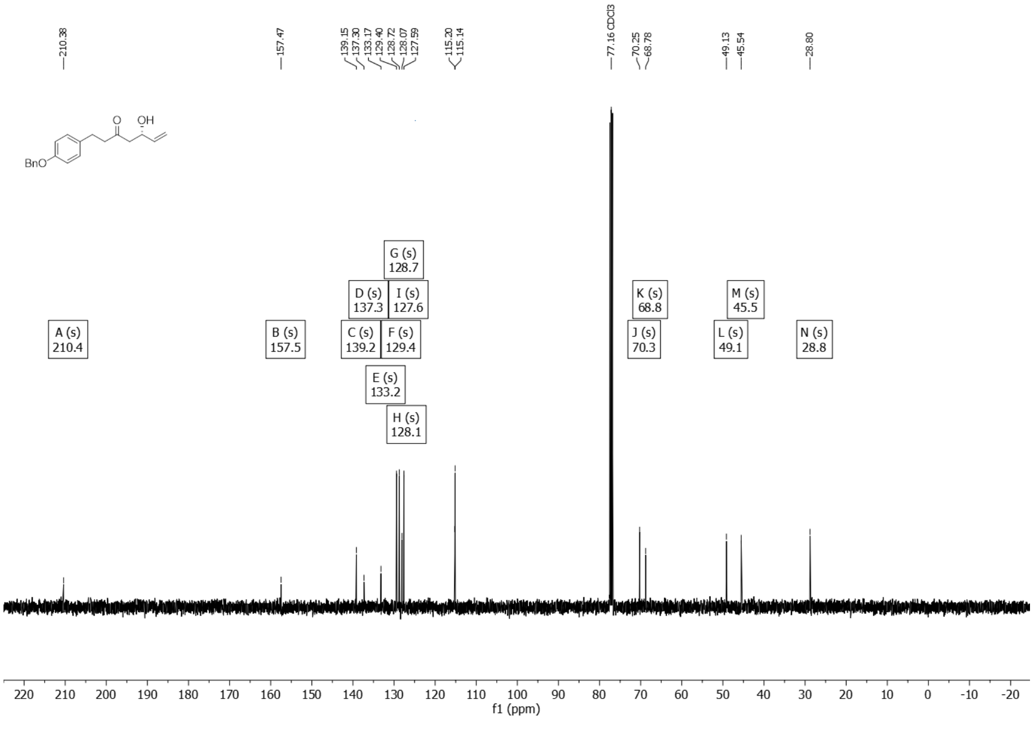

Acquisition Parameter

Source Type	ESI	Ion Polarity	Positive	Set Nebulizer	0.4 Bar
Focus	Not active			Set Dry Heater	200 °C
Scan Begin	50 m/z	Set Capillary	4500 V	Set Dry Gas	4.0 l/min
Scan End	1000 m/z	Set End Plate Offset	-500 V	Set Divert Valve	Waste

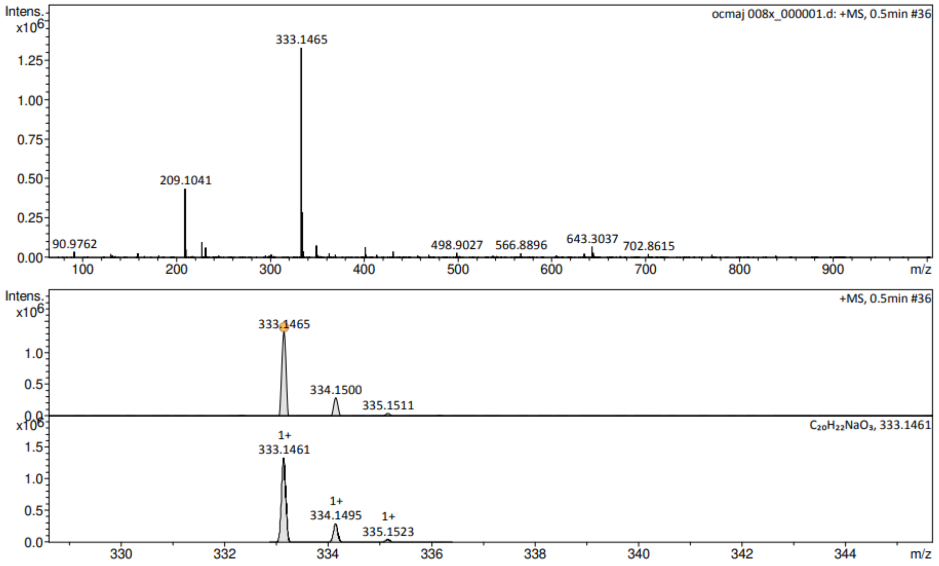

Meas. m/z	#	Ion Formula	m/z	err [ppm]	mSigma	# mSigma	Score	rdb	e⁻ Conf	N-Rule
333.1465	1	C20H22NaO3	333.1461	-1.2	2.3	1	100.00	9.5	even	ok

^{1}H, ^{13}C and HRMS spectra of (3S,5R)-7-(4-(benzyloxy)phenyl)hept-1-ene-3,5-diol (**16**)

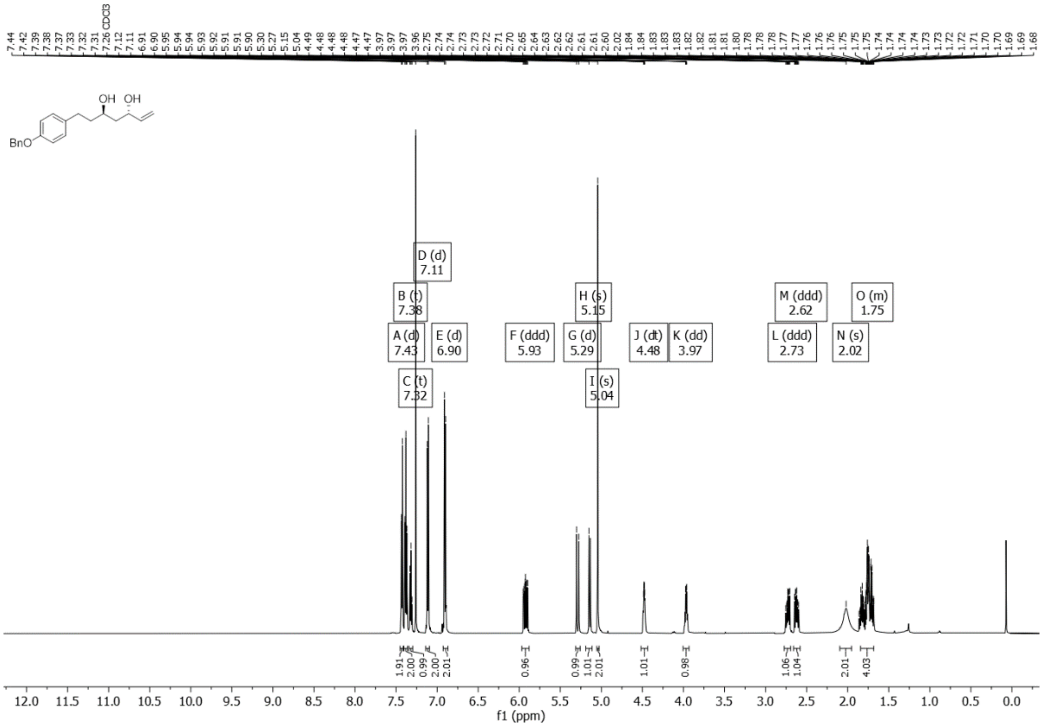

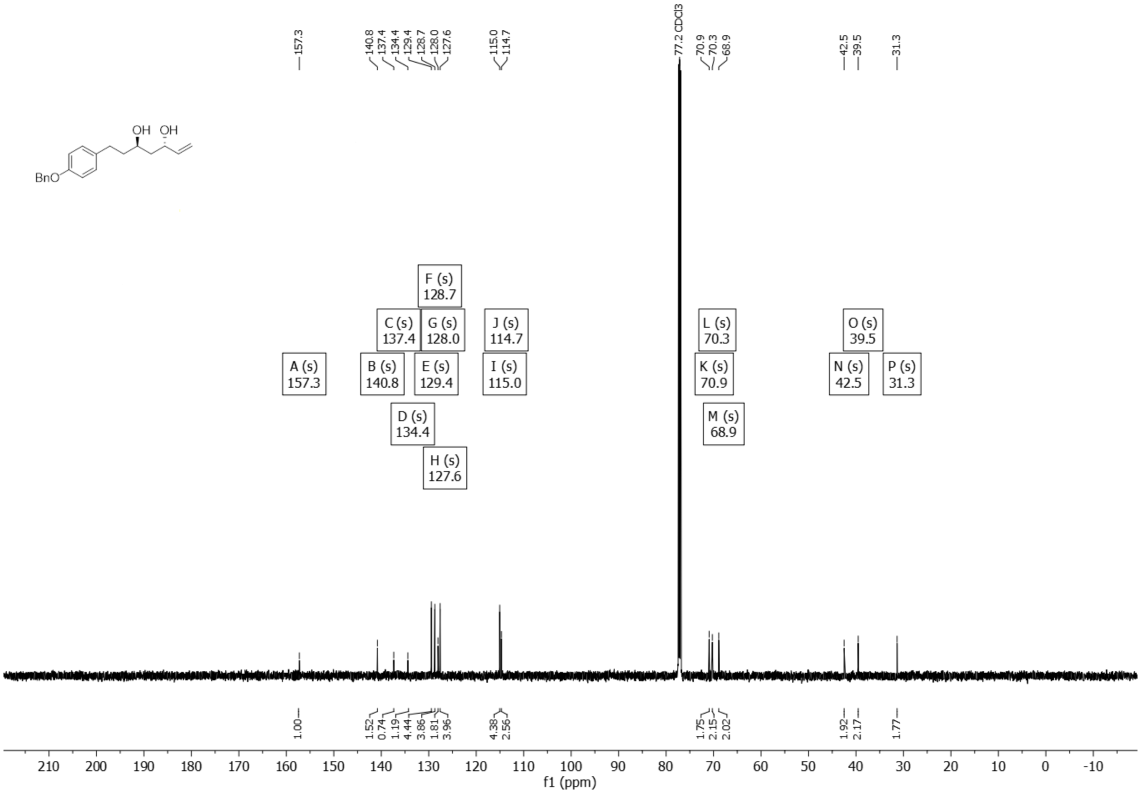

Acquisition Parameter

Source Type	ESI	Ion Polarity	Positive	Set Nebulizer	0.4 Bar
Focus	Not active			Set Dry Heater	200 °C
Scan Begin	50 m/z	Set Capillary	4500 V	Set Dry Gas	4.0 l/min
Scan End	1000 m/z	Set End Plate Offset	-500 V	Set Divert Valve	Waste

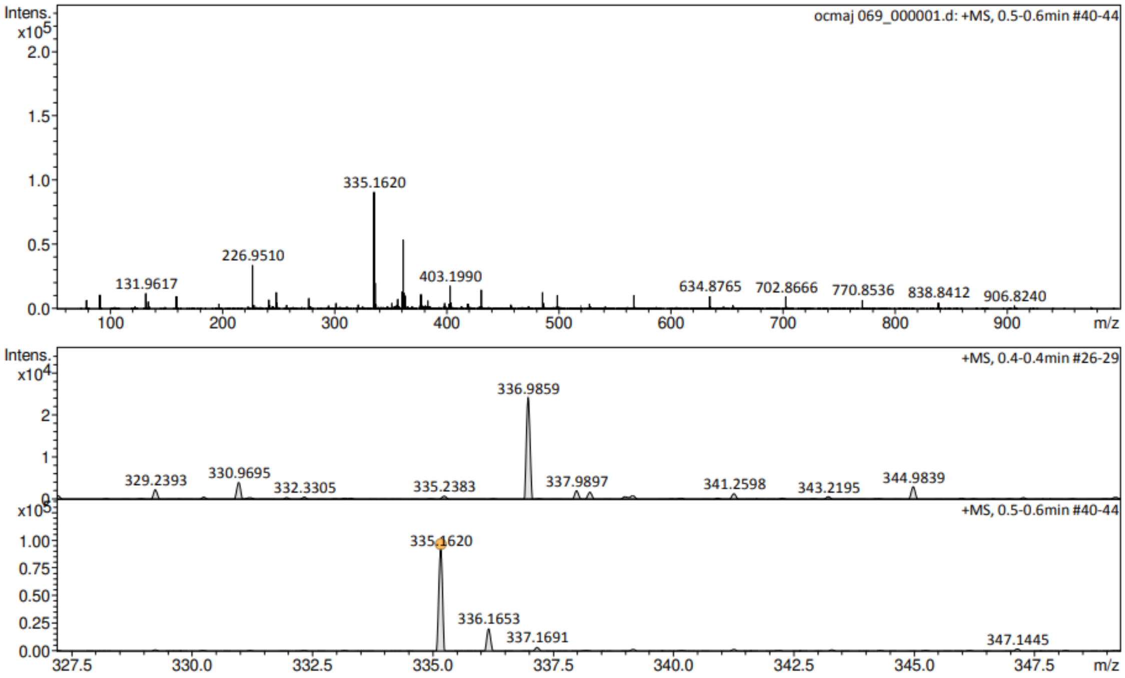

Meas. m/z	#	Ion Formula	m/z	err [ppm]	mSigma	# mSigma	Score	rdb	e⁻ Conf	N-Rule
335.1620	1	C20H24NaO3	335.1618	-0.7	2.2	1	100.00	8.5	even	ok

^{1}H, ^{13}C and HRMS spectra of ((((3S,5R)-7-(4-(benzyloxy)phenyl)hept-1-ene-3,5-diyl)bis(oxy))bis(methylene))dibenzene (**17**)

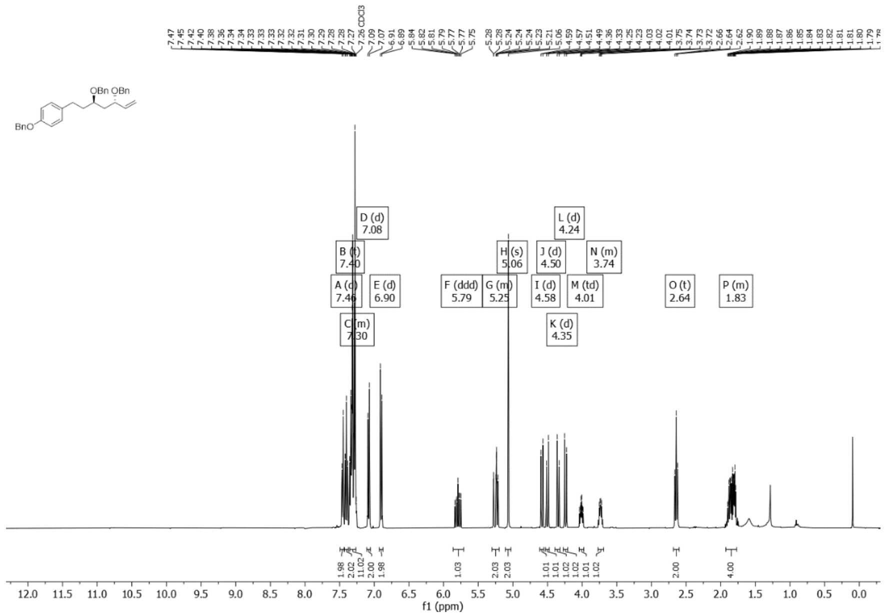

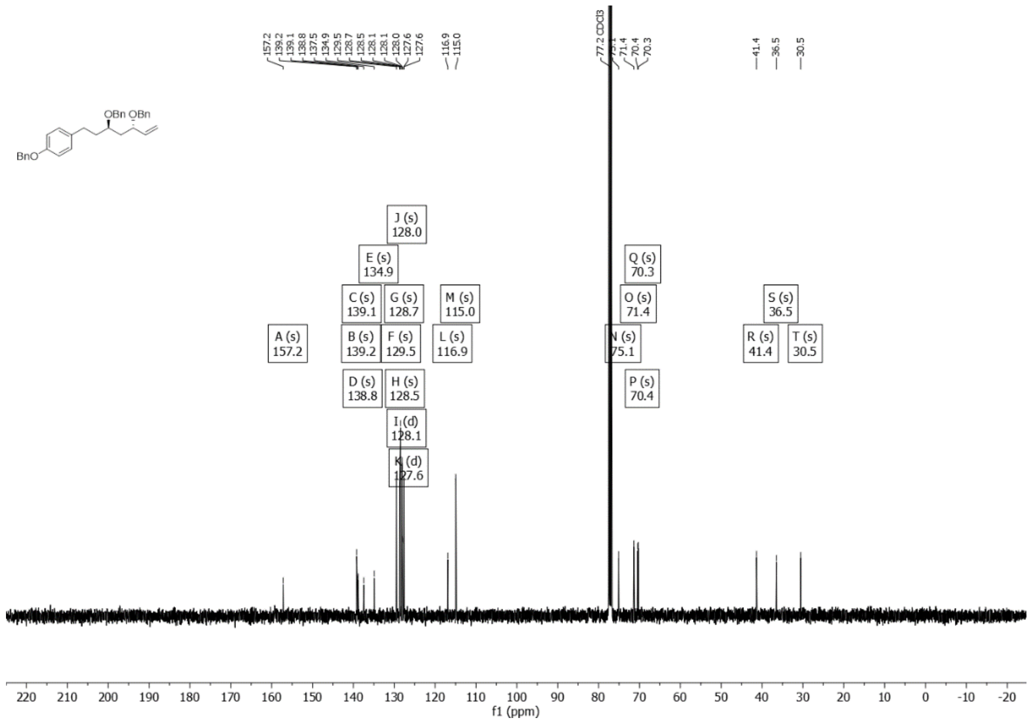

Acquisition Parameter

Source Type	ESI	Ion Polarity	Positive	Set Nebulizer	0.4 Bar
Focus	Not active			Set Dry Heater	200 °C
Scan Begin	50 m/z	Set Capillary	4500 V	Set Dry Gas	4.0 l/min
Scan End	1000 m/z	Set End Plate Offset	-500 V	Set Divert Valve	Waste

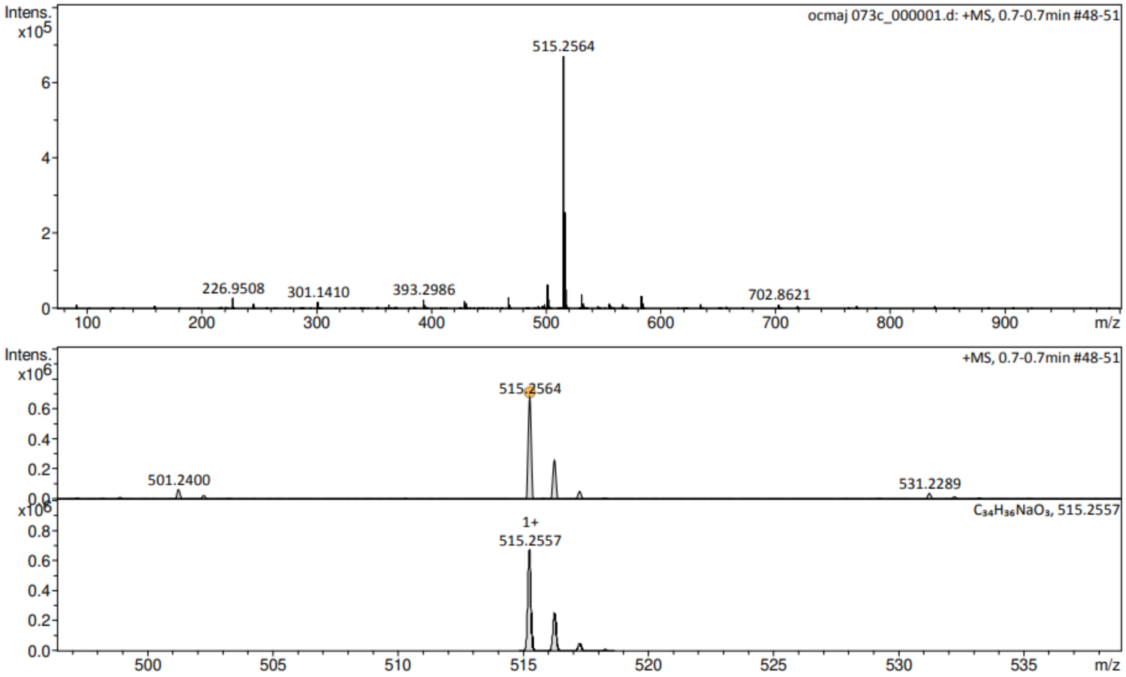

Meas. m/z	#	Ion Formula	m/z	err [ppm]	mSigma	# mSigma	Score	rdb	e⁻ Conf	N-Rule
515.2564	1	C34H36NaO3	515.2557	-1.4	3.9	1	100.00	16.5	even	ok

^{1}H, ^{13}C and HRMS spectra of 4,4'-((3S,5R,E)-3,5-bis(benzyloxy)hept-1-ene-1,7-diyl)bis((benzyloxy)benzene) (**19**)

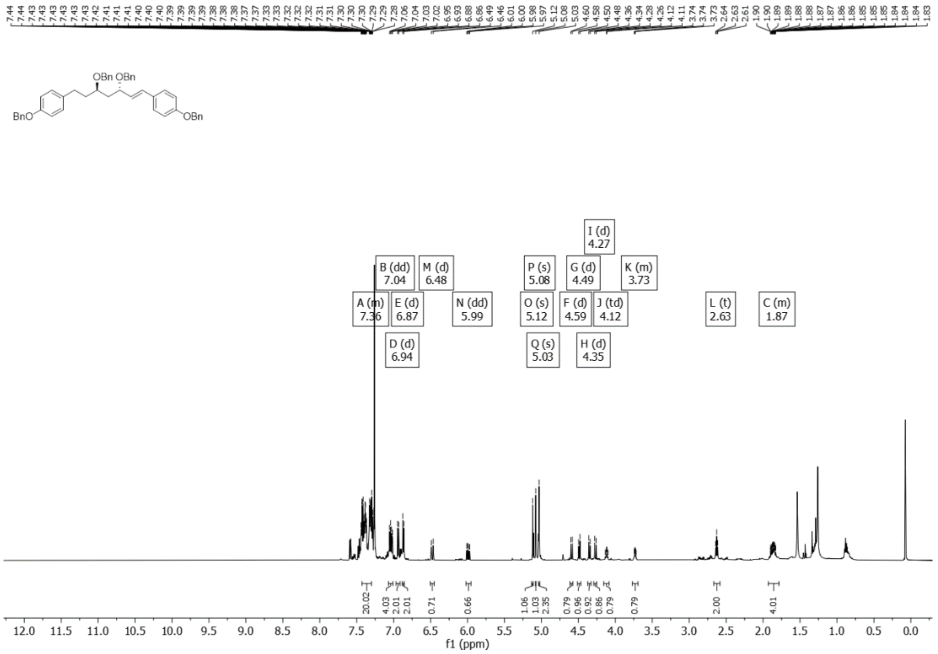

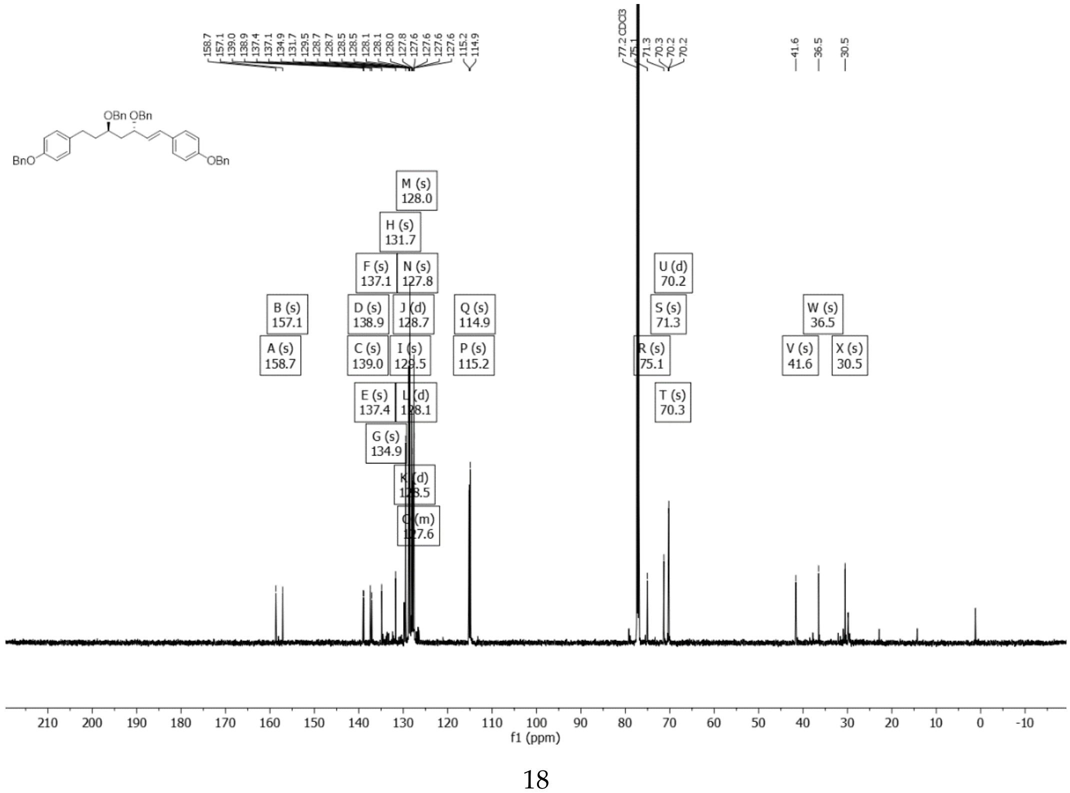

Acquisition Parameter

Source Type	ESI	Ion Polarity	Positive	Set Nebulizer	0.4 Bar
Focus	Not active			Set Dry Heater	200 °C
Scan Begin	50 m/z	Set Capillary	4500 V	Set Dry Gas	4.0 l/min
Scan End	1000 m/z	Set End Plate Offset	-500 V	Set Divert Valve	Waste

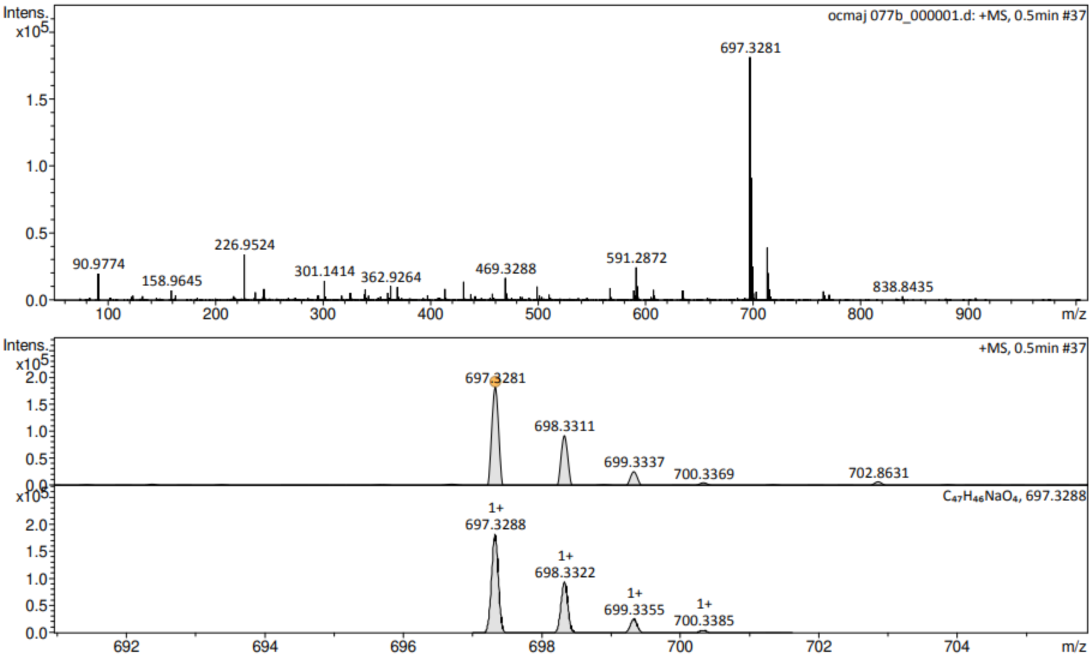

Meas. m/z	#	Ion Formula	m/z	err [ppm]	mSigma	# mSigma	Score	rdb	e⁻ Conf	N-Rule
697.3281	1	C47H46NaO4	697.3288	1.1	6.2	1	100.00	24.5	even	ok

^{1}H, ^{13}C and HRMS spectra of methyl 2-(4-methoxyphenyl)acetate (**21**)

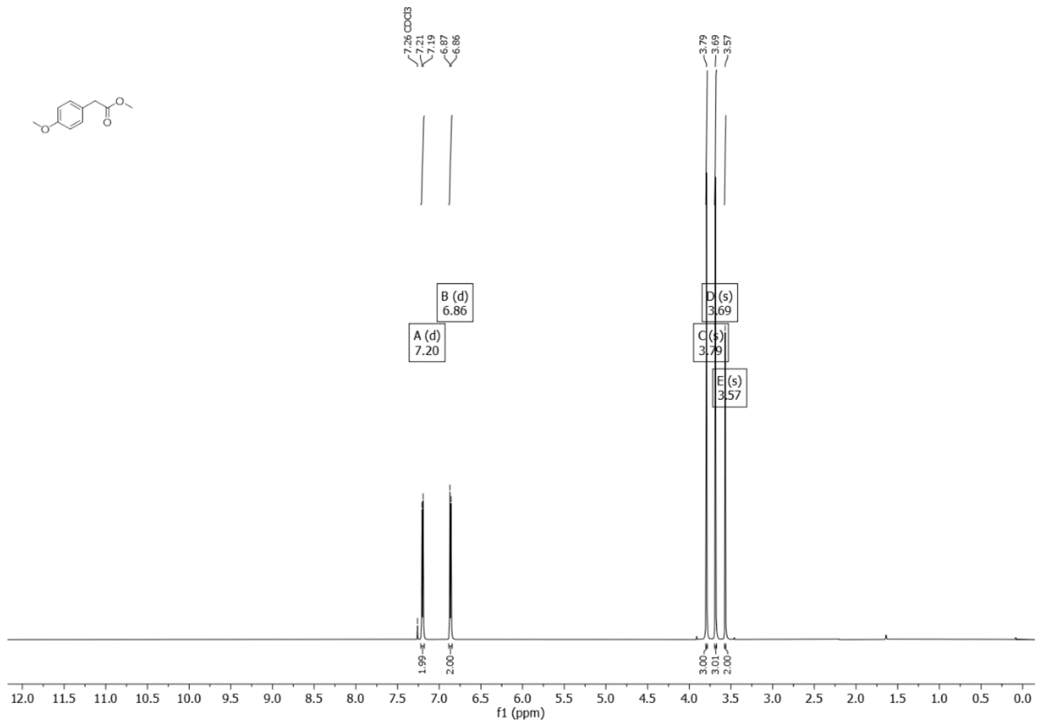

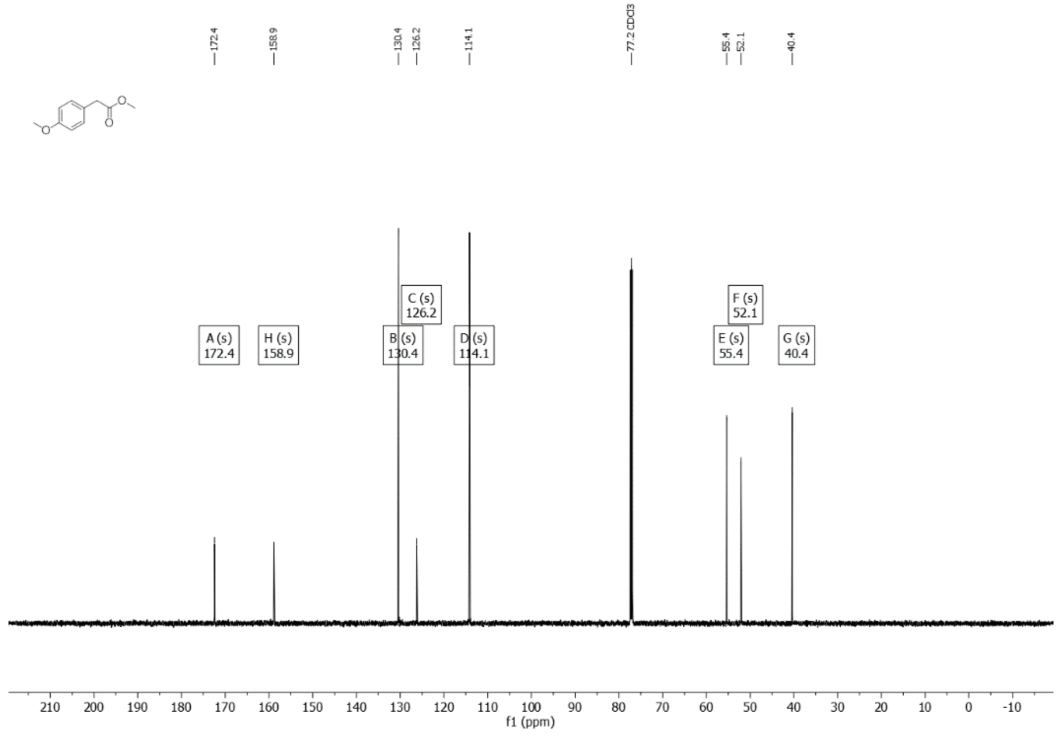

Acquisition Parameter

Source Type	ESI	Ion Polarity	Positive	Set Nebulizer	0.4 Bar
Focus	Not active			Set Dry Heater	200 °C
Scan Begin	50 m/z	Set Capillary	4500 V	Set Dry Gas	4.0 l/min
Scan End	1000 m/z	Set End Plate Offset	-500 V	Set Divert Valve	Waste

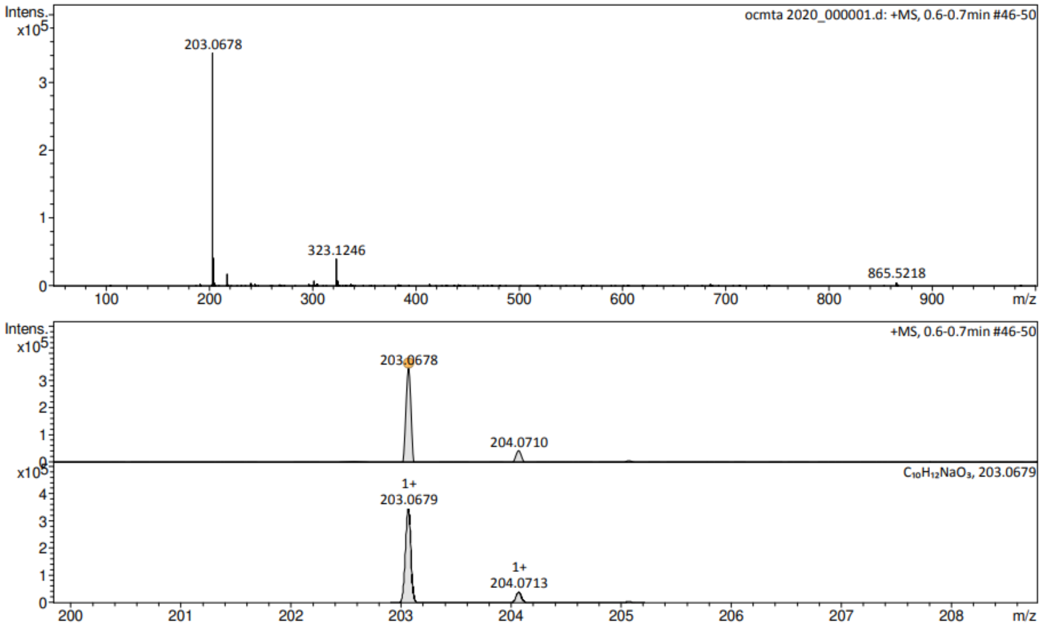

Meas. m/z	#	Ion Formula	m/z	err [ppm]	mSigma	# mSigma	Score	rdb	e⁻ Conf	N-Rule
203.0678	1	C10H12NaO3	203.0679	0.1	5.8	1	100.00	4.5	even	ok

^{1}H, ^{13}C and HRMS spectra of 2-(4-methoxyphenyl)acetaldehyde (**11**)

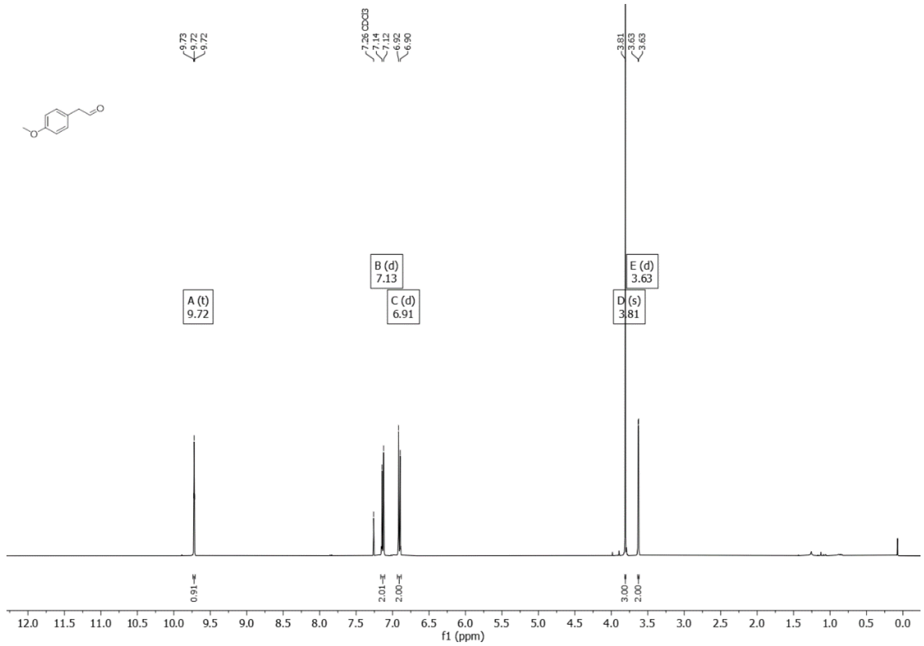

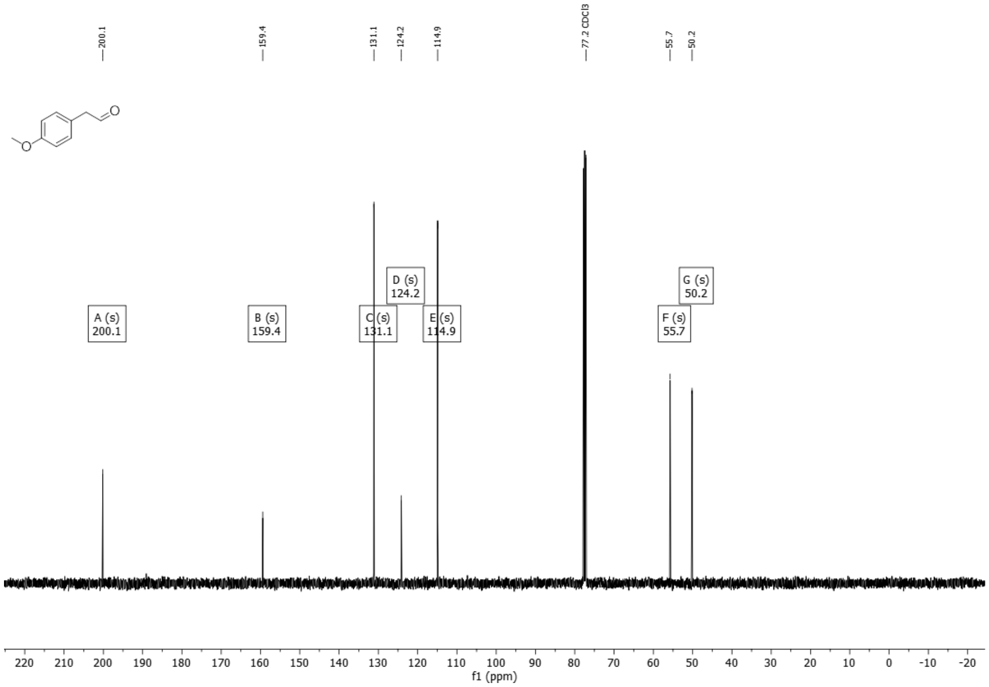

Acquisition Parameter

Source Type	ESI	Ion Polarity	Positive	Set Nebulizer	0.4 Bar
Focus	Not active			Set Dry Heater	200 °C
Scan Begin	50 m/z	Set Capillary	4500 V	Set Dry Gas	4.0 l/min
Scan End	1000 m/z	Set End Plate Offset	-500 V	Set Divert Valve	Waste

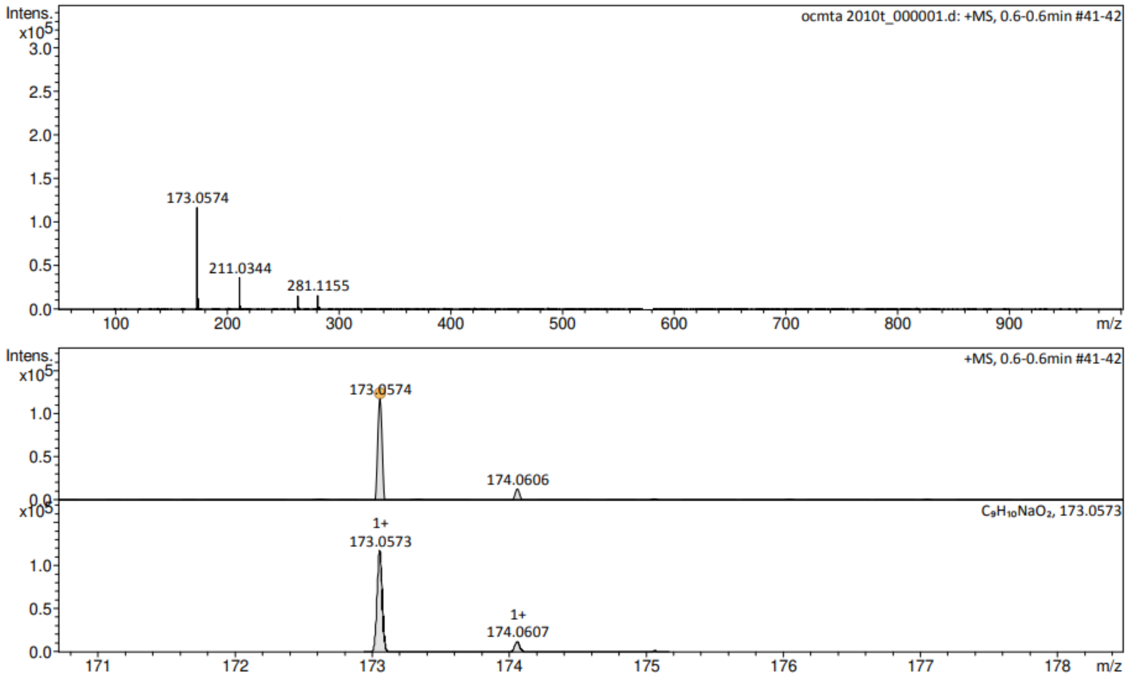

Meas. m/z	#	Ion Formula	m/z	err [ppm]	mSigma	# mSigma	Score	rdb	e⁻ Conf	N-Rule
173.0574	1	$C_9H_{10}NaO_2$	173.0573	-0.8	4.3	1	100.00	4.5	even	ok

^{1}H, ^{13}C and HRMS spectra of 5-hydroxy-1-(4-methoxyphenyl)hept-6-en-3-one (**22**)

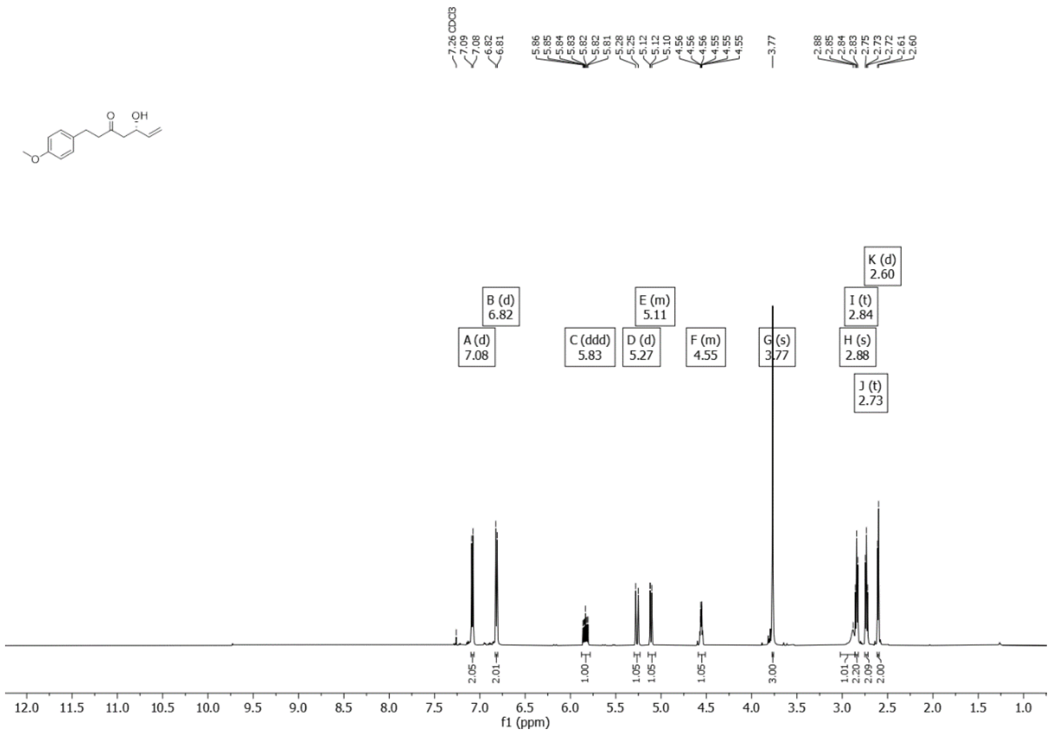

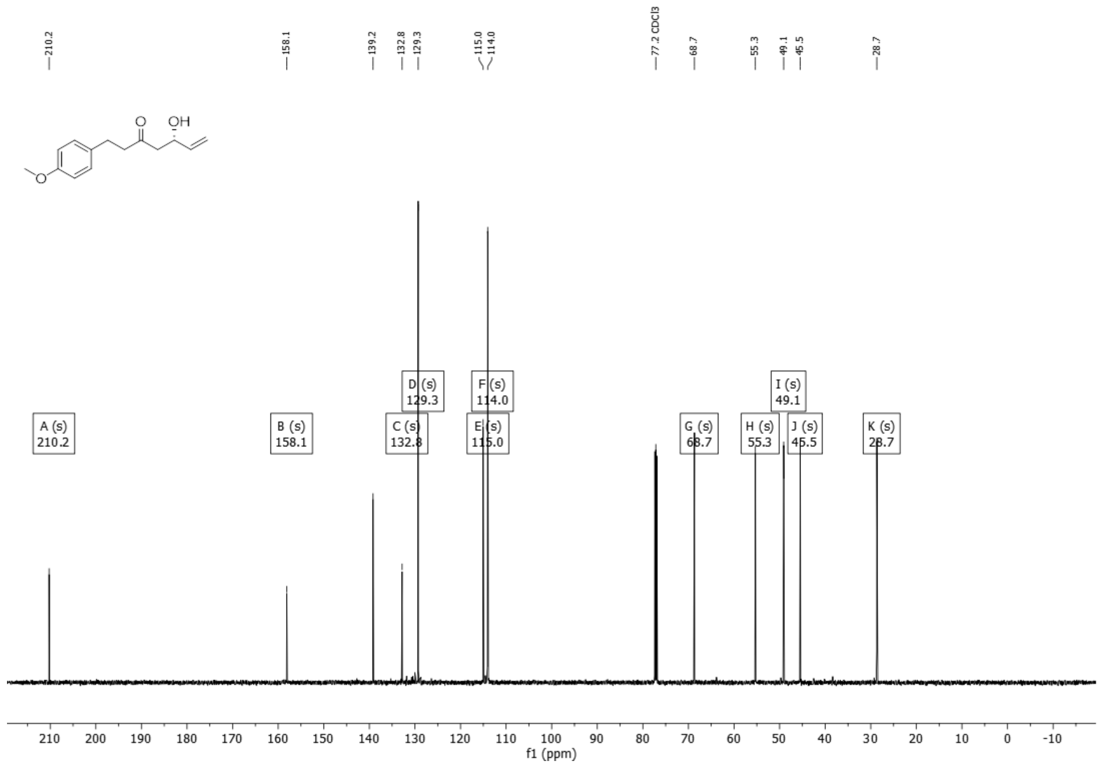

Acquisition Parameter

Source Type	ESI	Ion Polarity	Positive	Set Nebulizer	0.4 Bar
Focus	Not active			Set Dry Heater	200 °C
Scan Begin	50 m/z	Set Capillary	4500 V	Set Dry Gas	4.0 l/min
Scan End	1000 m/z	Set End Plate Offset	-500 V	Set Divert Valve	Waste

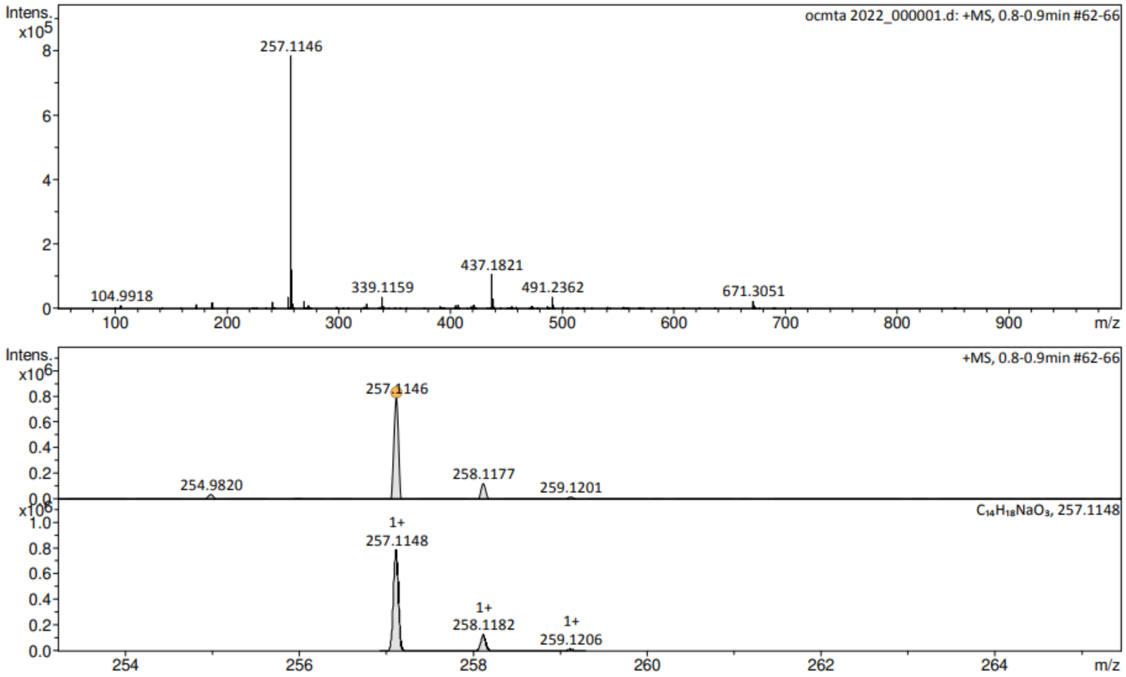

Meas. m/z	#	Ion Formula	m/z	err [ppm]	mSigma	# mSigma	Score	rdb	e⁻ Conf	N-Rule
257.1146	1	C14H18NaO3	257.1148	0.9	1.1	1	100.00	5.5	even	ok

^{1}H, ^{13}C and HRMS spectra of (3S,5R)-5-hydroxy-7-(4-methoxyphenyl)hept-1-en-3-yl acetate (**23a**)

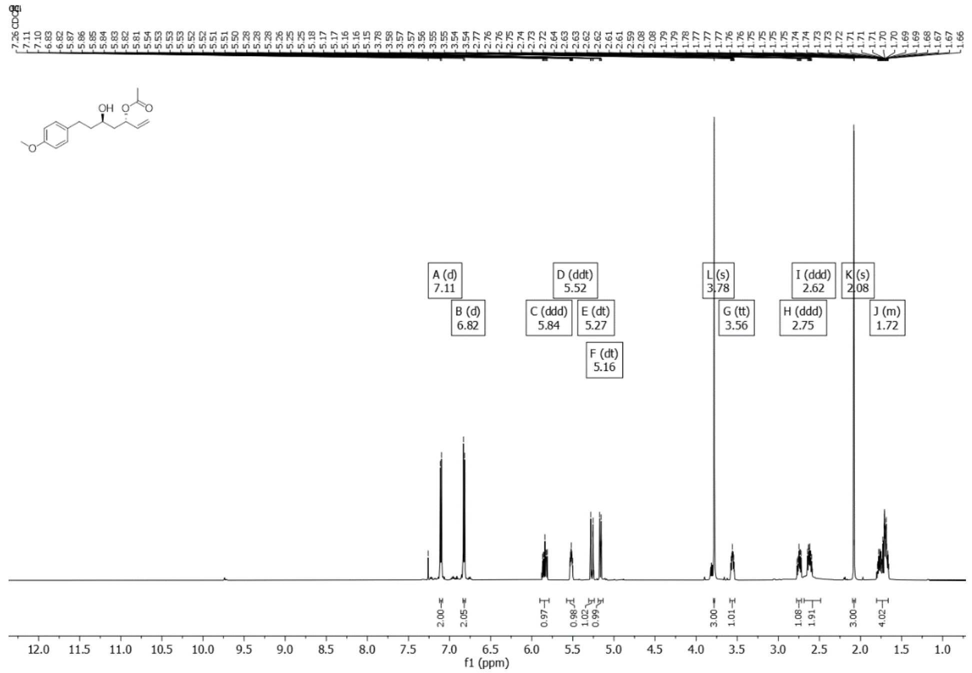

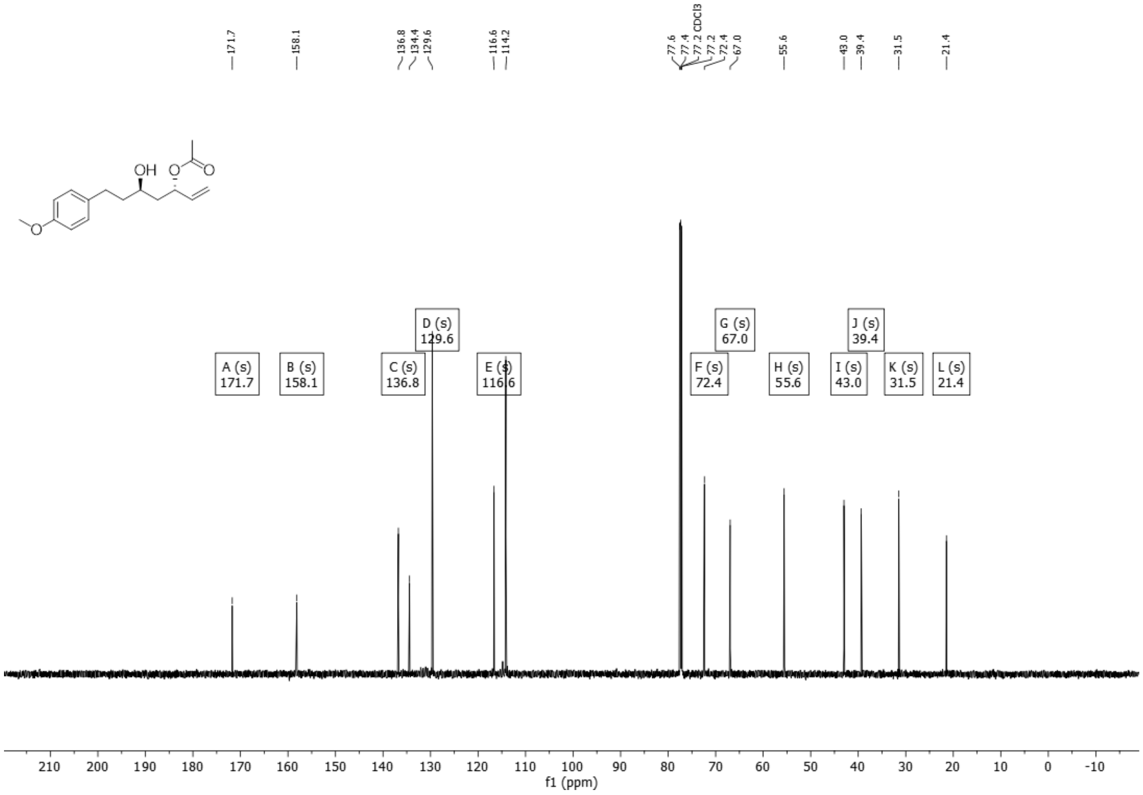

Acquisition Parameter

Source Type	ESI	Ion Polarity	Positive	Set Nebulizer	0.4 Bar
Focus	Not active			Set Dry Heater	200 °C
Scan Begin	50 m/z	Set Capillary	4500 V	Set Dry Gas	4.0 l/min
Scan End	1000 m/z	Set End Plate Offset	-500 V	Set Divert Valve	Waste

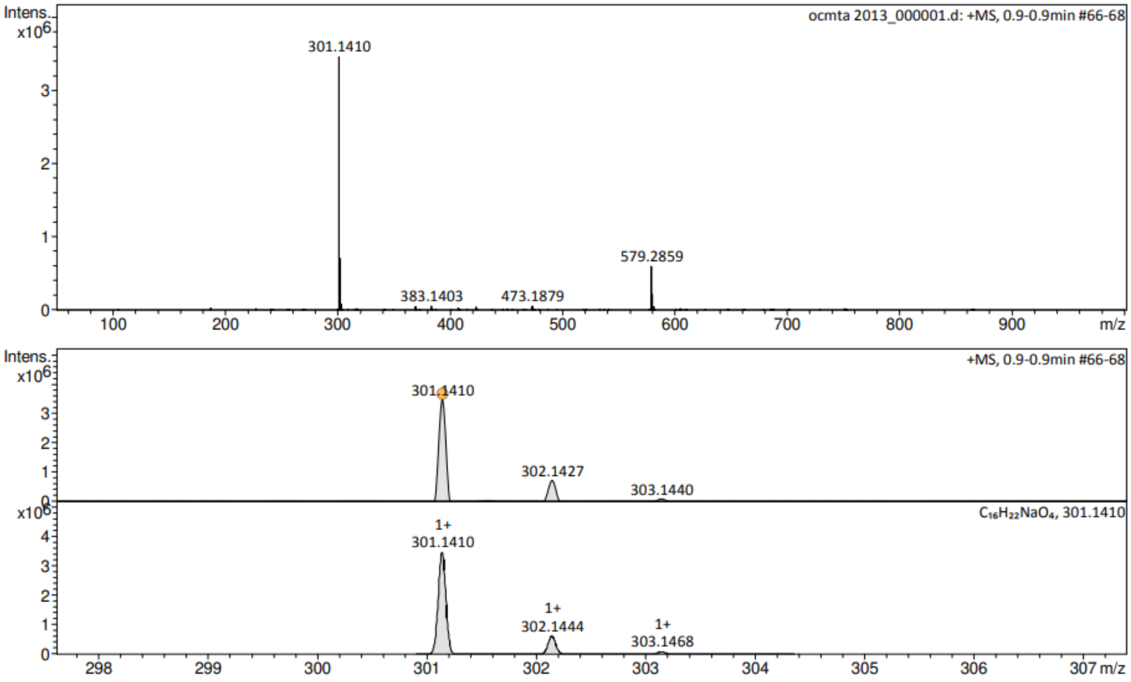

Meas. m/z	#	Ion Formula	m/z	err [ppm]	mSigma	# mSigma	Score	rdb	e⁻ Conf	N-Rule
301.1410	1	C16H22NaO4	301.1410	0.1	15.9	1	100.00	5.5	even	ok

^{1}H, ^{13}C and HRMS spectra of (3S,5R)-7-(4-methoxyphenyl)hept-1-ene-3,5-diol (**23**)

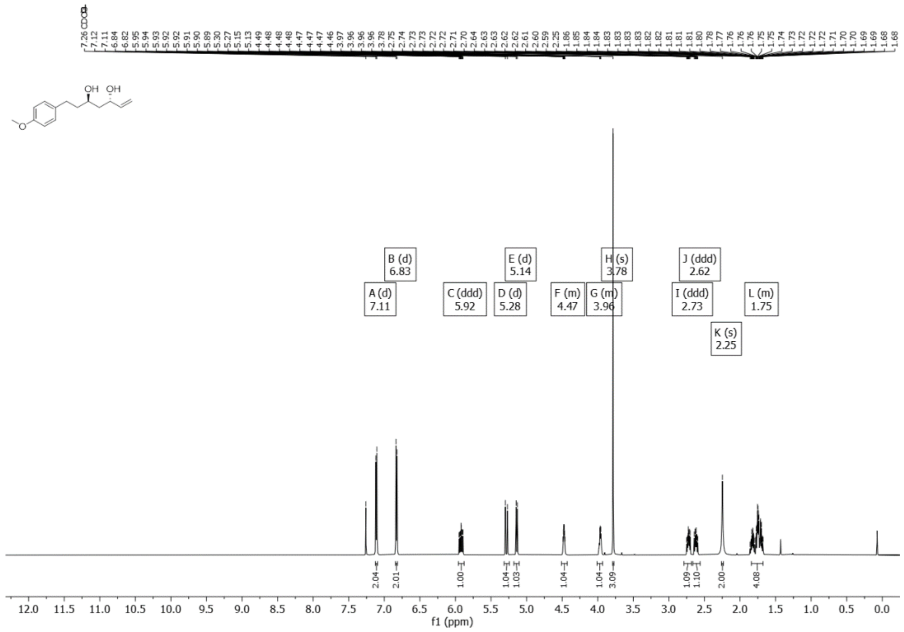

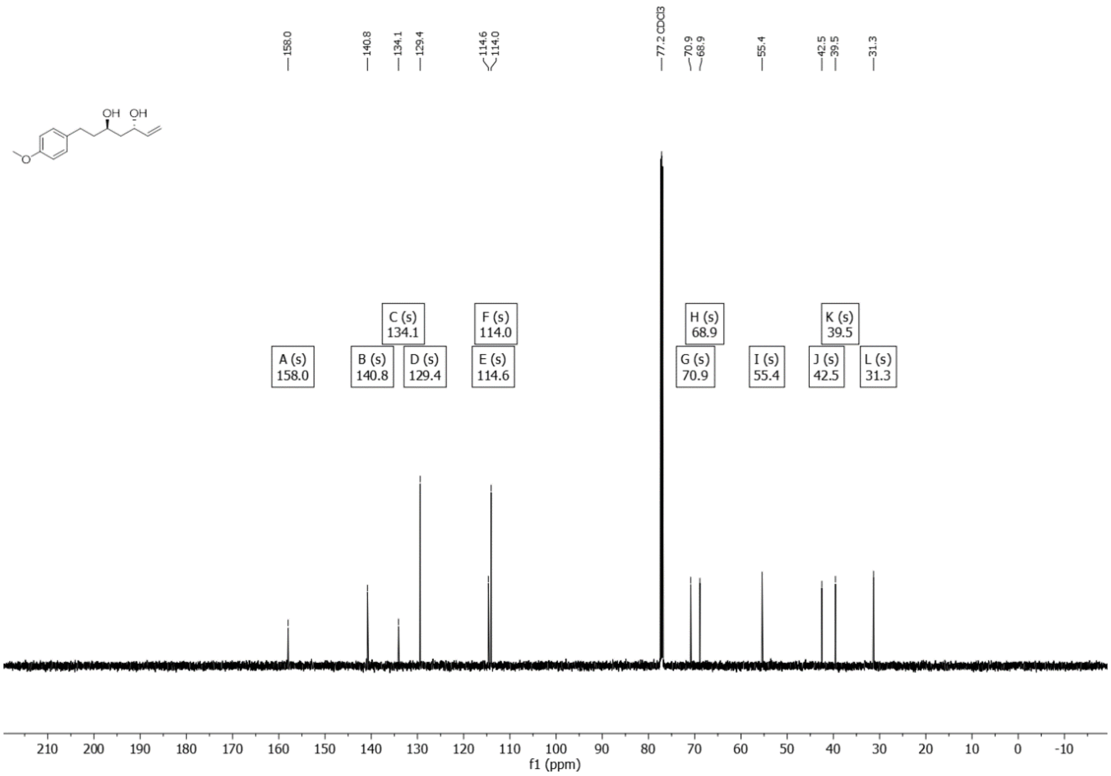

Acquisition Parameter

Source Type	ESI	Ion Polarity	Positive	Set Nebulizer	0.4 Bar
Focus	Not active			Set Dry Heater	200 °C
Scan Begin	50 m/z	Set Capillary	4500 V	Set Dry Gas	4.0 l/min
Scan End	1000 m/z	Set End Plate Offset	-500 V	Set Divert Valve	Waste

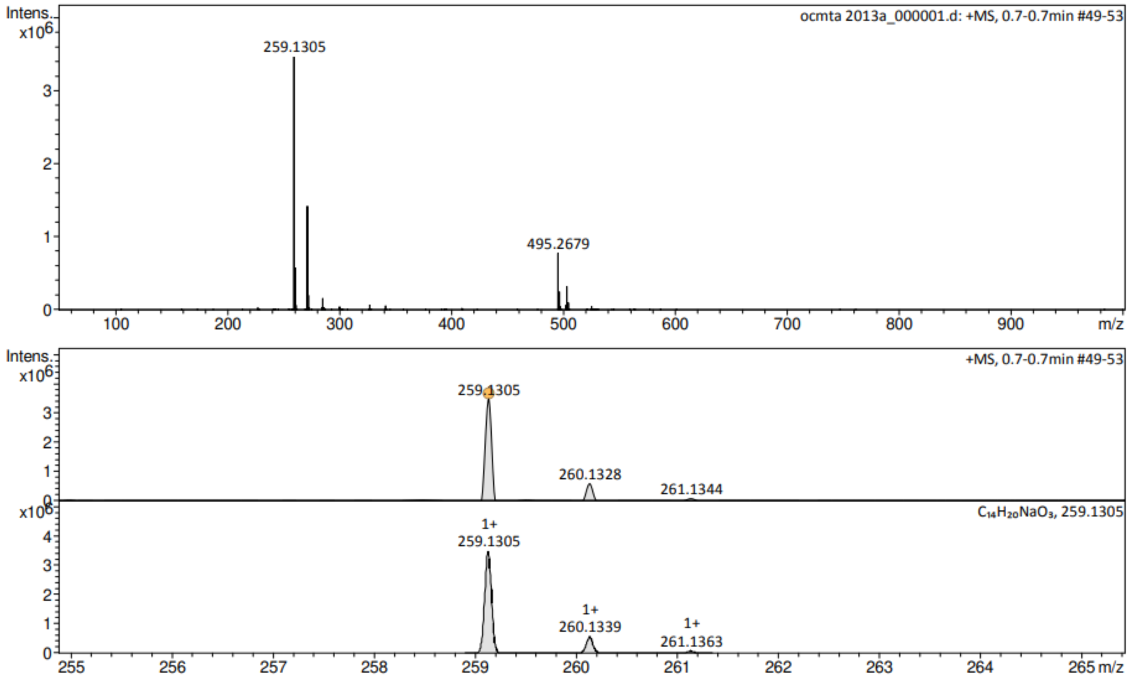

Meas. m/z	#	Ion Formula	m/z	err [ppm]	mSigma	# mSigma	Score	rdb	e⁻ Conf	N-Rule
259.1305	1	C14H20NaO3	259.1305	-0.2	6.7	1	100.00	4.5	even	ok

^{1}H, ^{13}C and HRMS spectra of (5R,7S)-5-(4-methoxyphenethyl)-2,2,3,3,9,9,10,10-octamethyl-7-vinyl-4,8-dioxa-3,9-disilaundecane (**24**)

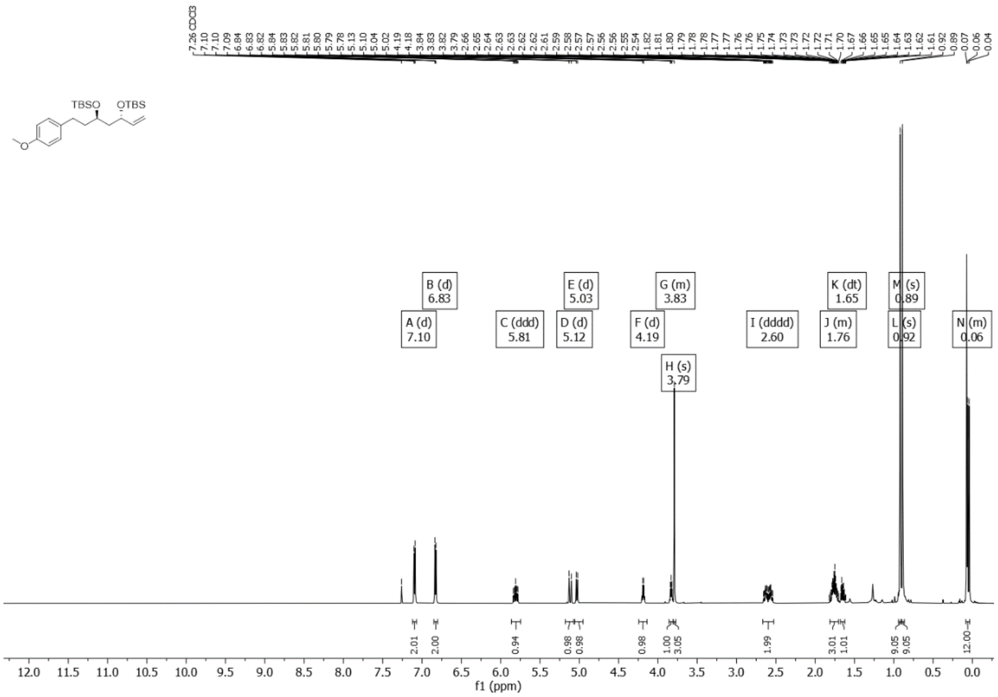

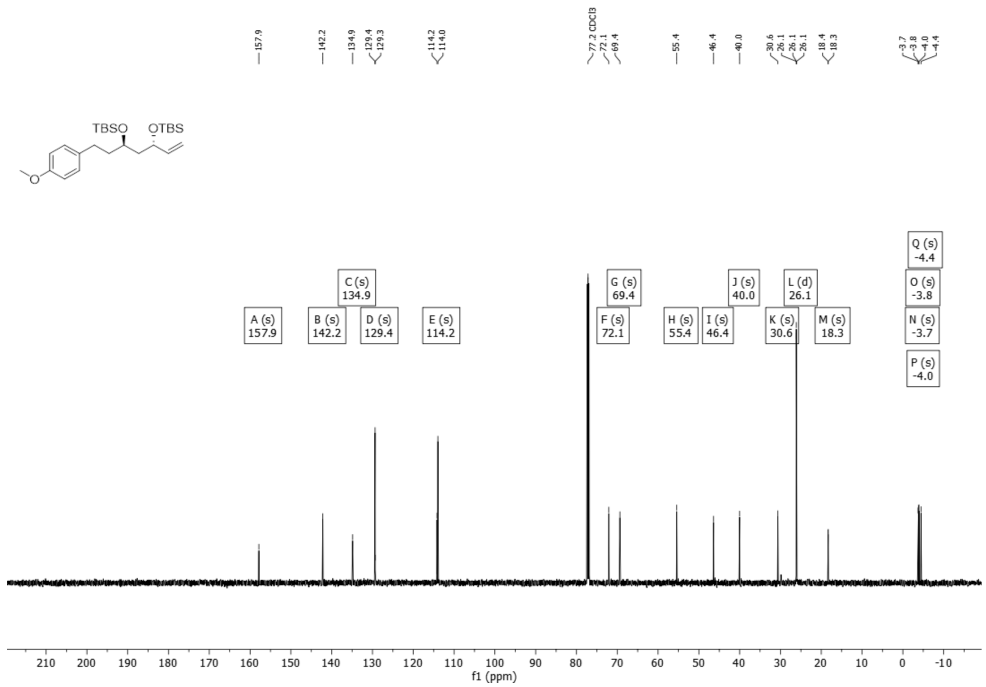

Acquisition Parameter

Source Type	ESI	Ion Polarity	Positive	Set Nebulizer	0.4 Bar
Focus	Not active			Set Dry Heater	200 °C
Scan Begin	50 m/z	Set Capillary	4500 V	Set Dry Gas	4.0 l/min
Scan End	1000 m/z	Set End Plate Offset	-500 V	Set Divert Valve	Waste

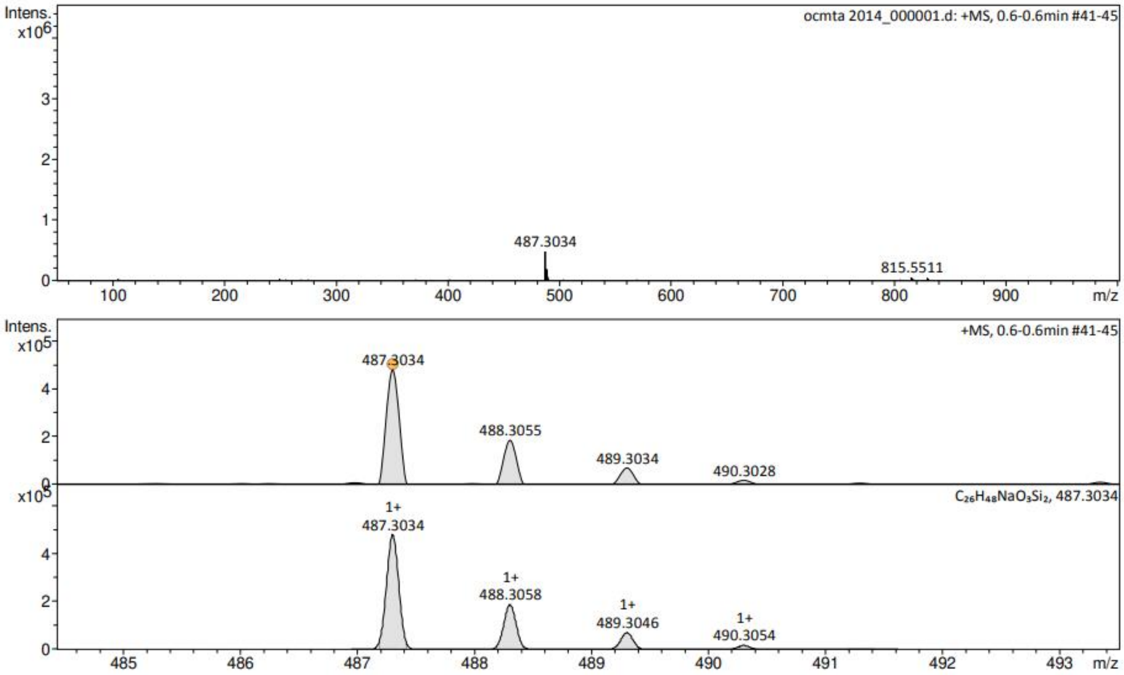

Meas. m/z	#	Ion Formula	m/z	err [ppm]	mSigma	# mSigma	Score	rdb	e⁻ Conf	N-Rule
487.3034	1	$C_{26}H_{48}NaO_3Si_2$	487.3034	0.0	2.2	1	100.00	4.5	even	ok

^{1}H, ^{13}C and HRMS spectra of (5R,7S)-5-(4-methoxyphenethyl)-7-((E)-4-methoxystyryl)-2,2,3,3,9,9,10,10-octamethyl-4,8-dioxa-3,9-disilaundecane (**26**)

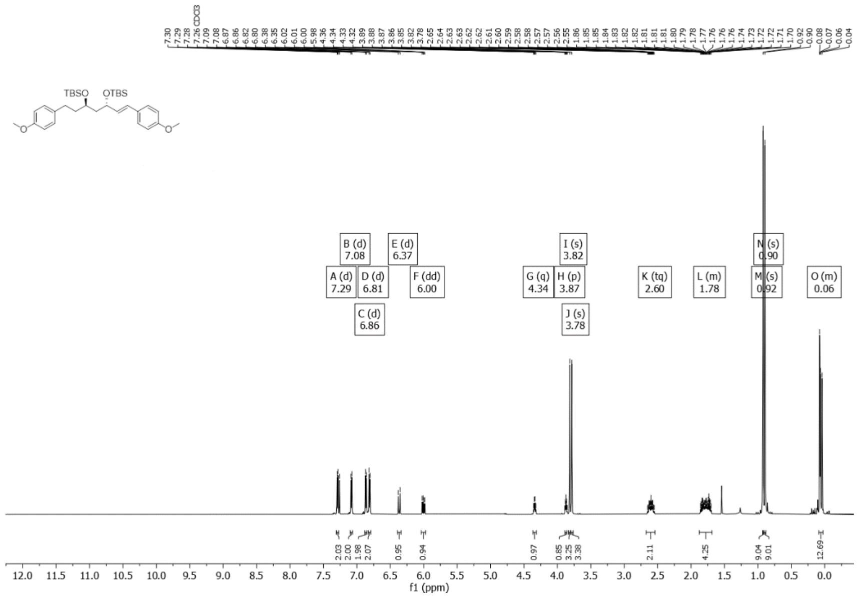

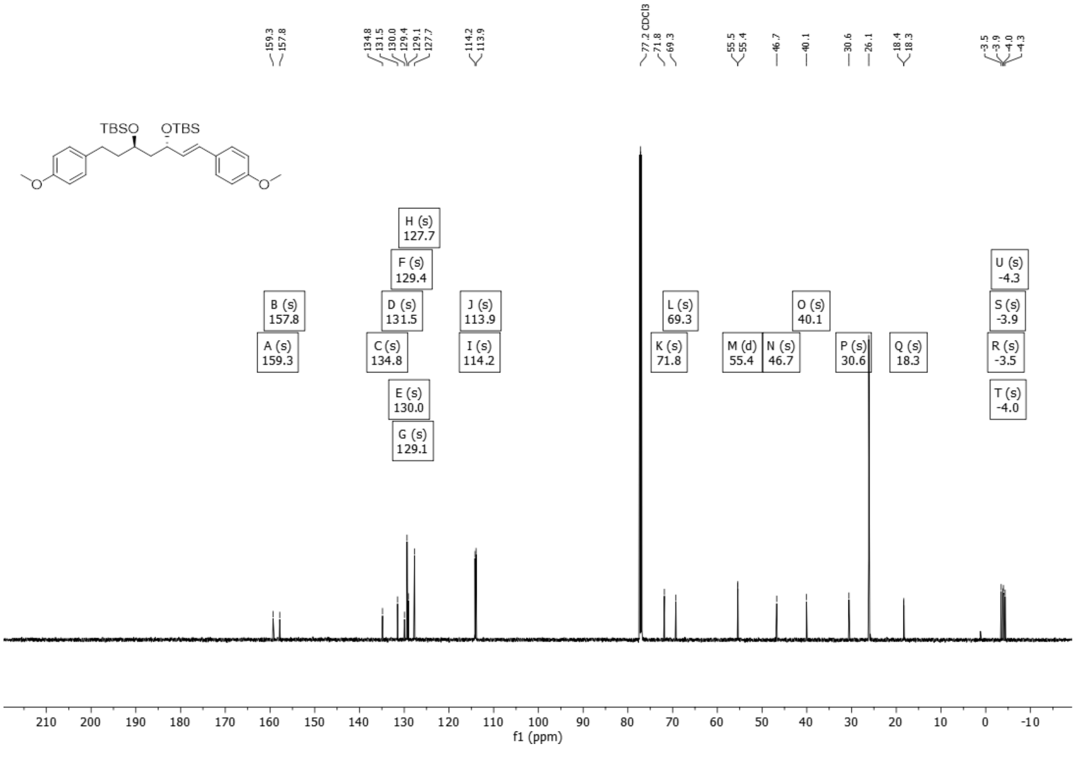

Acquisition Parameter

Source Type	ESI	Ion Polarity	Positive	Set Nebulizer	0.4 Bar
Focus	Not active			Set Dry Heater	200 °C
Scan Begin	50 m/z	Set Capillary	4500 V	Set Dry Gas	4.0 l/min
Scan End	1000 m/z	Set End Plate Offset	-500 V	Set Divert Valve	Waste

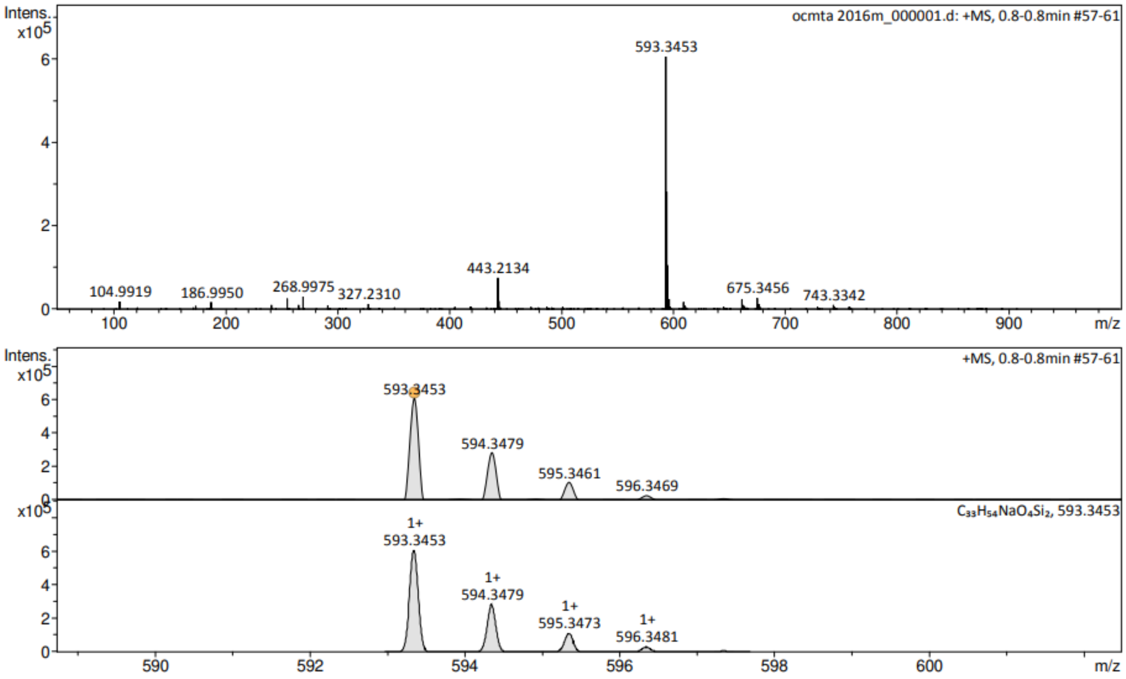

Meas. m/z	#	Ion Formula	m/z	err [ppm]	mSigma	# mSigma	Score	rdb	e⁻ Conf	N-Rule
593.3453	1	C33H54NaO4Si2	593.3453	0.1	4.6	1	100.00	8.5	even	ok

^{1}H and ^{13}C spectra of (5R,7R)-5,7-bis(4-Methoxyphenethyl)-2,2,3,3,9,9,10,10-octamethyl-4,8-dioxa-3,9-disilaundecane (**27**)

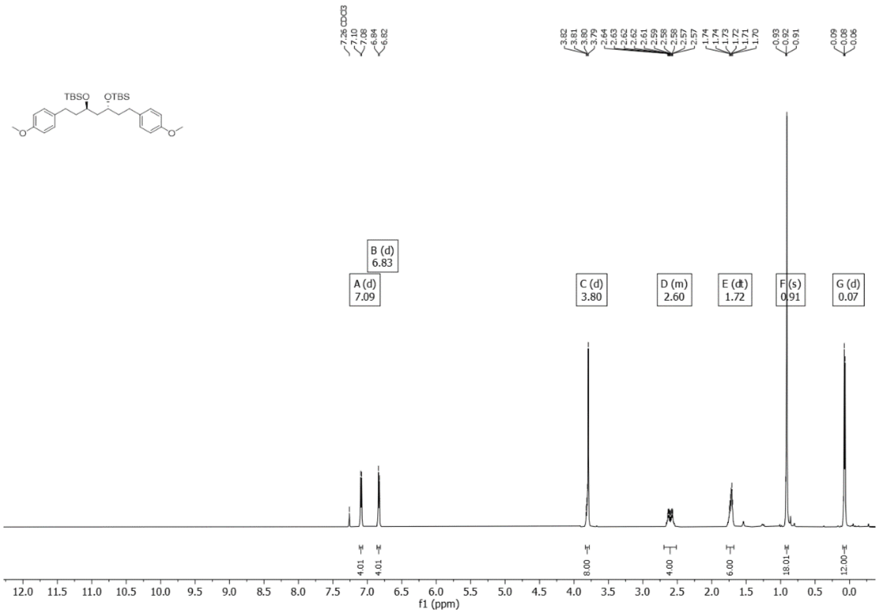

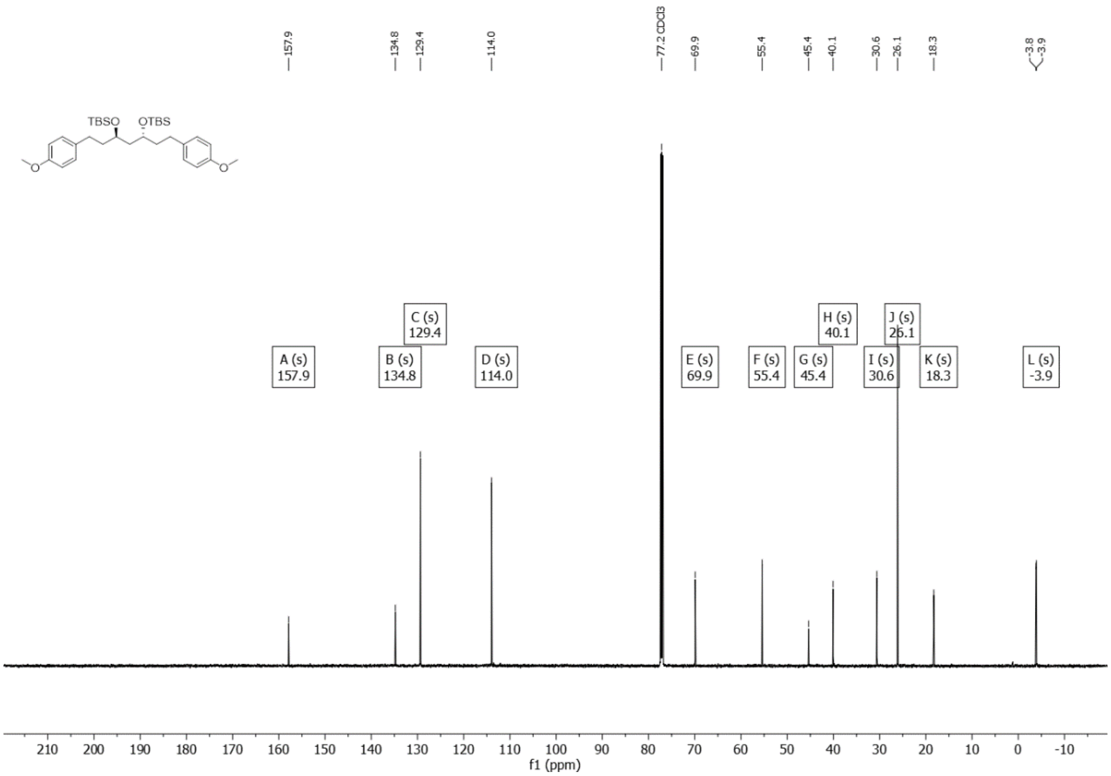

Acquisition Parameter

Source Type	ESI	Ion Polarity	Positive	Set Nebulizer	0.4 Bar
Focus	Not active			Set Dry Heater	200 °C
Scan Begin	50 m/z	Set Capillary	4500 V	Set Dry Gas	4.0 l/min
Scan End	1000 m/z	Set End Plate Offset	-500 V	Set Divert Valve	Waste

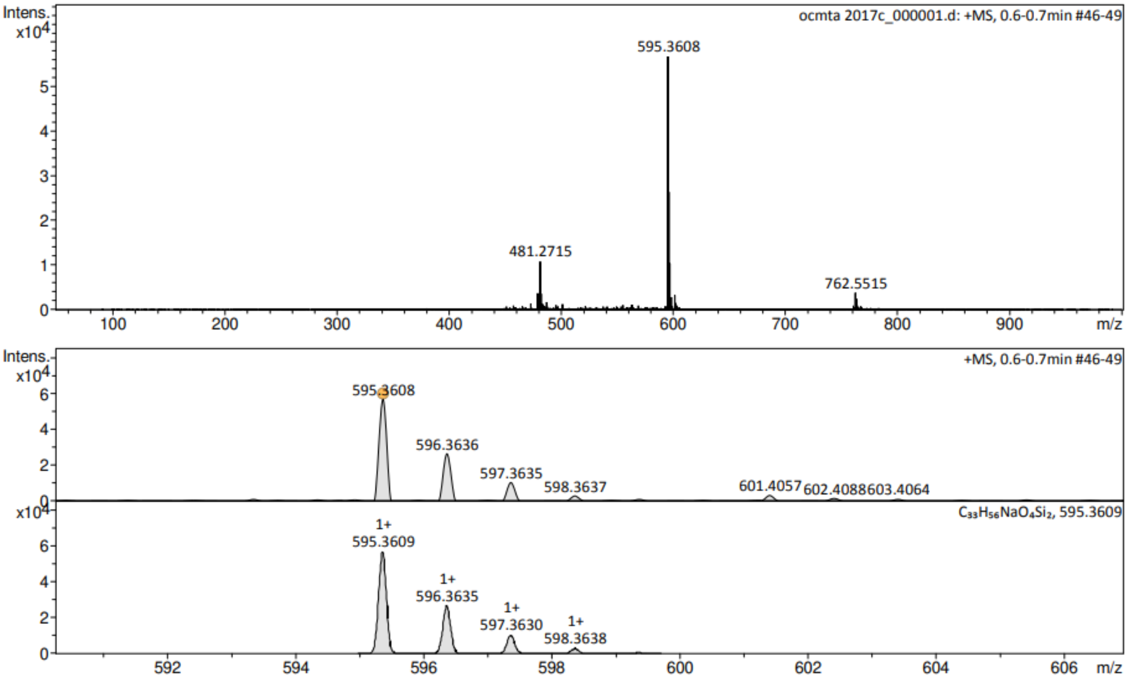

Meas. m/z	#	Ion Formula	m/z	err [ppm]	mSigma	# mSigma	Score	rdb	e⁻ Conf	N-Rule
595.3608	1	C33H56NaO4Si2	595.3609	0.2	2.6	1	100.00	7.5	even	ok

^{1}H and ^{13}C spectra of (3R,5R)-1,7-bis(4-methoxyphenyl)heptane-3,5-diol (**4**)

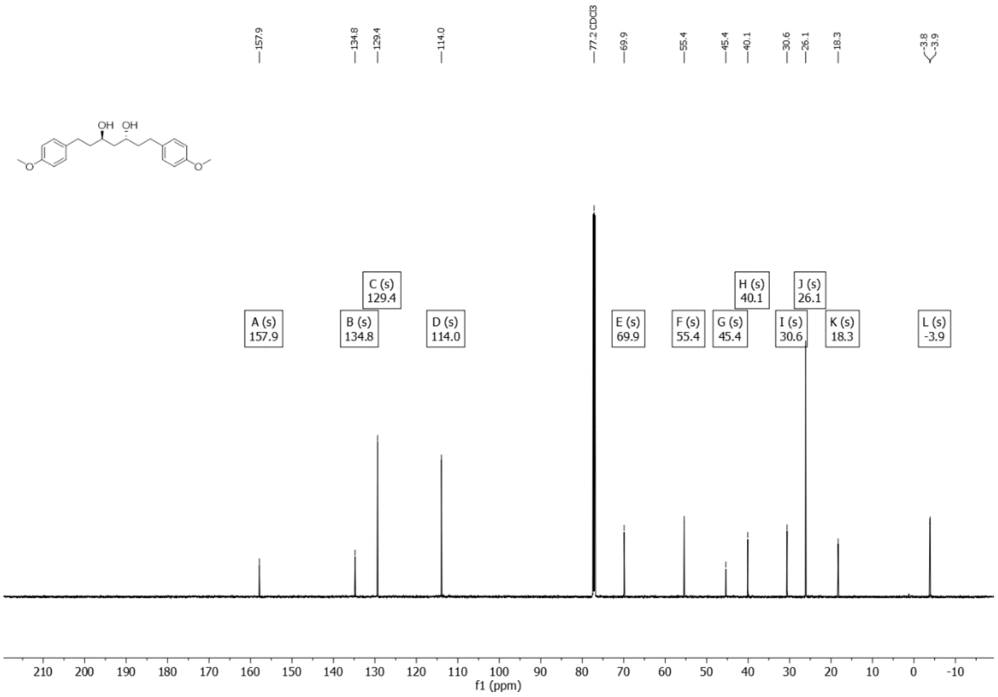

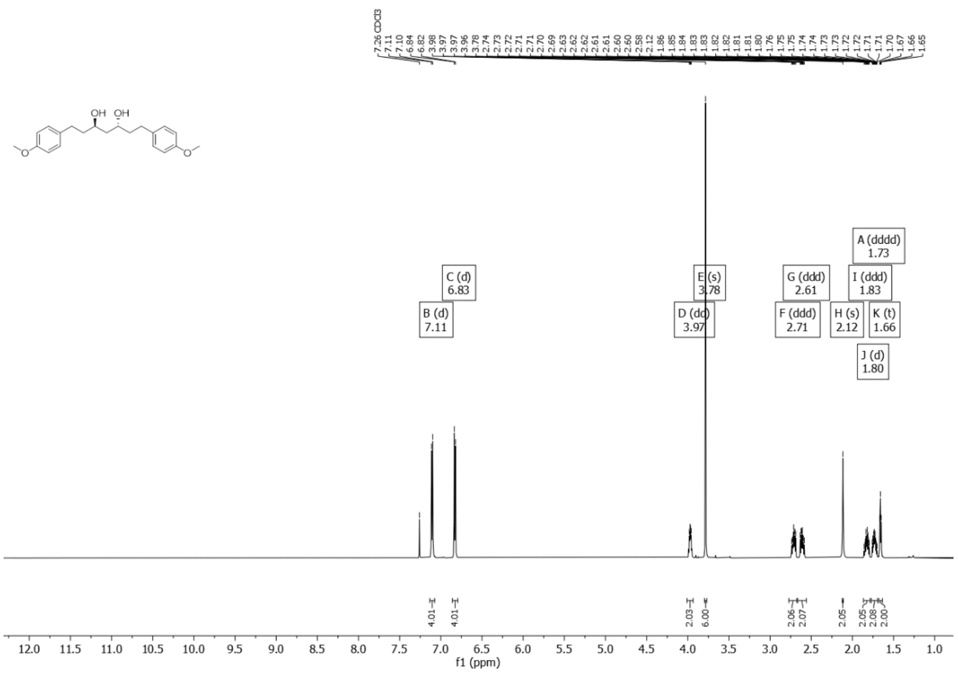

Acquisition Parameter

Source Type	ESI	Ion Polarity	Positive	Set Nebulizer	0.4 Bar
Focus	Not active			Set Dry Heater	200 °C
Scan Begin	50 m/z	Set Capillary	4500 V	Set Dry Gas	4.0 l/min
Scan End	1000 m/z	Set End Plate Offset	-500 V	Set Divert Valve	Waste

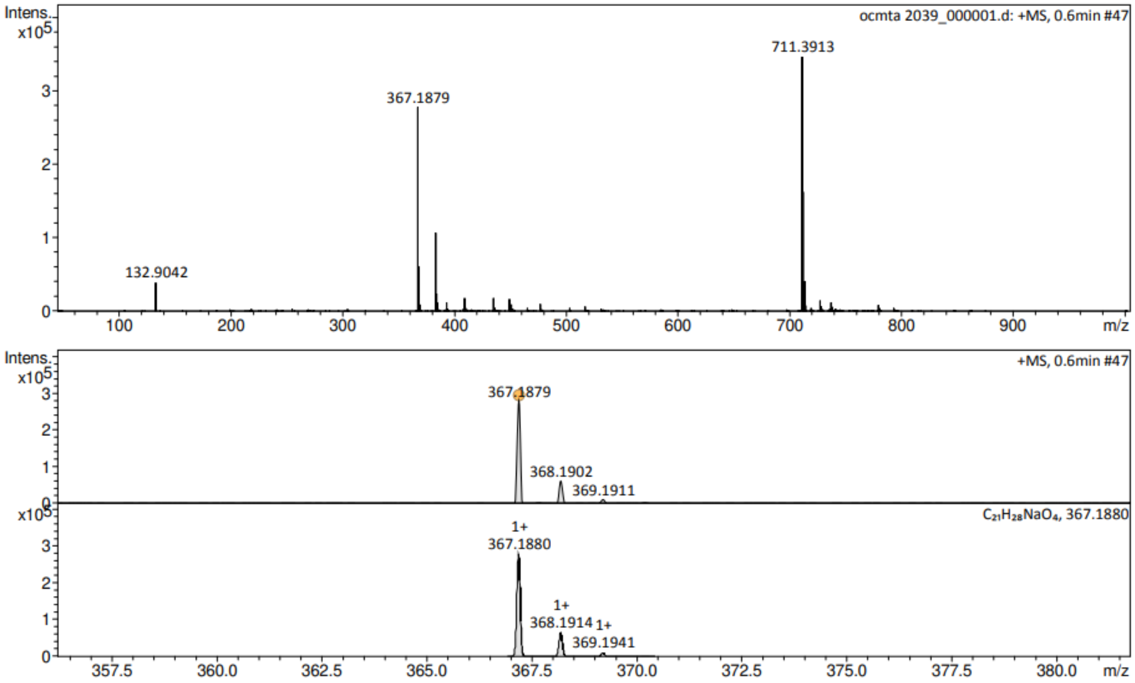

Meas. m/z	#	Ion Formula	m/z	err [ppm]	mSigma	# mSigma	Score	rdb	e⁻ Conf	N-Rule
367.1879	1	$C_{21}H_{28}NaO_4$	367.1880	0.3	8.9	1	100.00	7.5	even	ok
